# Effects of variable magnetic field and partial slips on the dynamics of Sutterby nanofluid due to biaxially exponential and nonlinear stretchable sheets

**DOI:** 10.1016/j.heliyon.2023.e17921

**Published:** 2023-07-05

**Authors:** Bushra Ishtiaq, Sohail Nadeem, Jehad Alzabut

**Affiliations:** a Department of Mathematics, Quaid-I-Azam University, 45320, Islamabad, 44000, Pakistan; b Department of Mathematics and Sciences, Prince Sultan University, 11586, Riyadh, Saudi Arabia; c Department of Industrial Engineering, OSTIM Technical University, Ankara, 06374, Turkey

**Keywords:** Sutterby nanofluid, Variable magnetic field, Stretchable exponential sheet, Partial slips, Buongiorno model, Stretchable nonlinear sheet

## Abstract

Based on both the characteristics of shear thinning and shear thickening fluids, the Sutterby fluid has various applications in engineering and industrial fields. Due to the dual nature of the Sutterby fluid, the motive of the current study is to scrutinize the variable physical effects on the Sutterby nanofluid flow subject to shear thickening and shear thinning behavior over biaxially stretchable exponential and nonlinear sheets. The steady flow mechanism with the variable magnetic field, partial slip effects, and variable heat source/sink is examined over both stretchable sheets. The analysis of mass and heat transfer is carried out with the mutual impacts of thermophoresis and Brownian motion through the Buongiorno model. Suitable transformations for both exponential and nonlinear sheets are implemented on the problem's constitutive equations. As a result, the nonlinear setup of ordinary differential equations is acquired which is further numerically analyzed through the bvp4c technique in MATLAB. The graphical explanation of temperature, velocity, and concentration distributions exhibits that the exponential sheet provides more significant results as compared to the nonlinear sheet. Further, this study revealed that for the shear thickening behavior of Sutterby nanofluid, the increasing values of Deborah number increase the axial velocity.

## Nomenclature

Cartesian coordinates (m): $\tilde{X},\tilde{Y},\tilde{Z}$
Thermophoresis parameter: Nt
Reference length (m): $\tilde{L}$
Heat flux: $q_{w}$
Identity tensor: I
Prandtl number: Pr
Velocity slip parameters along $\tilde{X}$ and $\tilde{Y}$ directions: $\xi_{1},\xi_{2}$
Zero shear rate viscosity (Pas): $\mu_{0}$
Sheet velocity in $\tilde{Y}$ direction $\left(\frac{m}{s}\right)$: ${\tilde{V}}_{sh}$
Specific heat capacity $\left(J\;{kg}^{-1}K^{-1}\right)$: $c_{p}$
Local Reynolds number along $\tilde{Y}$ direction: ${Re}_{\tilde{Y}}$
Local Nusselt number: ${Nu}_{\tilde{X}}$
Magnetic field parameter: M
Velocity field: V
Fluid density (kgm3): ρ
Sheet temperature (K): ${\tilde{T}}_{sh}$
Local Sherwood number: ${Sh}_{\tilde{X}}$
Velocity slip factors along $\tilde{X}$ and $\tilde{Y}$ directions: $N_{1}^{*},N_{2}^{*}$
Ambient fluid concentration $\left(kgm^{-3}\right)$: ${\tilde{C}}_{\infty }$
Ratio of nanoparticle heat capacity and base fluid heat capacity: τ
Sheet velocity in $\tilde{X}$ direction $\left(\frac{m}{s}\right)$: ${\tilde{U}}_{sh}$
Extra stress tensor: S
Concentration exponent: $\beta^{*}$
Fluid thermal conductivity $\left(\frac{W}{mk}\right)$: k
Sherwood number: ${Sh}_{x}$
Volumetric rate of heat source/sink $\left(Wm^{-2}K^{-1}\right)$: $Q_{0}$
Cauchy stress tensor: T
Mass flux: $q_{m}$
Local Nusselt number: ${Nu}_{x}$
Ambient fluid temperature (K): ${\tilde{T}}_{\infty }$
Power-law index of nonlinear sheet: $M^{*}$
Temperature exponent: $\alpha^{*}$
Material constant: β
Reynolds number: Re
Fluid pressure (Pa): p
Skin friction coefficients along $\tilde{X}$ and $\tilde{Y}$ directions: ${Cf}_{\tilde{X}},{Cf}_{\tilde{Y}}$
Kinematic viscosity $\left(\frac{m^{2}}{s}\right)$: υ
Positive constants: ${\tilde{U}}_{0},{\tilde{V}}_{0}$
Thermophoretic diffusion coefficient: $D_{T}$
Shear stresses: $\tau_{\tilde{Z}\tilde{X}},\tau_{\tilde{Z}\tilde{Y}}$
Deborah number: De
First Rivlin-Erickson tensor: A
Schmidt number: Sc
Velocity components $\left(\frac{m}{s}\right)$: $\tilde{U},\tilde{V},\tilde{W}$
Stretching ratio parameter: δ
Constant strength of magnetic field (kgs2): $B_{0}$
Heat source/sink parameter: $Q^{*}$
Power-law index: N
Local Reynolds number along $\tilde{X}$ direction: ${Re}_{\tilde{X}}$
Constants: $A_{1},A_{2}$
Brownian diffusion coefficient: $D_{B}$
Electrical conductivity ${\left(\Omega m\right)}^{-1}$: σ
Thermal diffusivity $\left(\frac{m^{2}}{s}\right)$: α
Brownian motion parameter: Nb

## 1 Introduction

Non-Newtonian fluids gained significant importance due to a wide range of their practices in the fields of engineering and biology. Implementations of such fluids are paper production, oil supplies, cooling system, food preparation, colloidal suspensions, nuclear reactors, etc. Non-Newtonian fluids have complexity in their mathematical structures. Thus, a single constitutive equation is not sufficient to deliberate all their characteristics. To overcome this drawback, various models of non-Newtonian fluids based on the three categories (pseudoplastic/shear thinning, thixotropic, dilatant/shear thickening) have been proposed. The Sutterby fluid model, which demonstrates both the properties of shear thickening fluid and shear thinning fluid relative to the different ranges of the power-law index, is one of the significant non-Newtonian fluid models. The model of Sutterby fluid was first proposed by Sutterby [1]. Due to its dual nature, this fluid model has significant importance for many researchers. Ahmad et al. [2] worked on the Sutterby fluid to investigate its radiative flow mechanism within the parallel disks. They looked at how the double stratification affected the mechanics of heat and mass transfer. The two-dimensional radiative flow phenomenon developed by a porous surface in Sutterby fluid with nanoparticles was demonstrated by Bouslimi et al. [3]. The radiative flow behavior of a magnetized Sutterby nanofluid was studied by Shahzad et al. [4] subject to a sloping sheet. With the consideration of a stretchable surface, Jamshed et al. [5] inspected the consequences of thermal radiation and activation energy on the flow behavior of a Sutterby hybrid nanofluid. Through a stretchable exponential medium, Bouslimi et al. [6] inspected the time-independent magnetized flow mechanism developed in a Sutterby nanofluid. With the involvement of gyrotactic microbes, the bioconvection flow phenomenon of Sutterby fluid was studied by Abdal et al. [7]. Rehman et al. [8] discussed the magnetized flow behavior of Sutterby nanofluid and examined the heat transfer mechanism with heat flux theory.

The involvement of the small-sized nanoparticles (oxides, metals, carbon nanotubes) in the traditional fluid (ethylene glycol, water, oil) boosts the base fluid's thermal conductivity. The high efficiency of the tiny nanoparticles augmented the ability of the heat transfer in the base fluid. The contribution of the individual nanoparticle to the base fluid yields the nanofluid. The volume fraction and size of the nanoparticles affect the thermal conductivity of the nanofluid. In various fields, nanofluid has beneficial practices such as home appliances cooling/heating, electronics, transportation, chemical procedures equipment solar energy, and vehicle thermal management. The slip mechanisms between the base fluid and nanoparticle were initially studied by Buongiorno [9]. After briefly analyzing these mechanisms, Buongiorno proposed the two-phase model with the contribution of the two mechanisms namely thermophoresis and Brownian diffusion. After that, various researchers discussed the Buongiorno model for flow problems with numerous physical circumstances. Khan et al. [10] scrutinized the axisymmetric hydromagnetic flow phenomenon with the collaboration of the two-phase Buongiorno model in a Sisko nanofluid. The 2D flow phenomenon of second-grade nanofluid with the significance of the Buongiorno model and physical impacts was studied by Gangadhar et al. [11]. They examined that the flow velocity deteriorates corresponding to the improved fluid parameter. Gangadhar et al. [12] also explored the magnetized flow behavior of Oldroyd- B nanofluid originating from a vertical surface with the association of the Buongiorno model. Ishtiaq and Nadeem [13] discussed the flow behavior of Casson fluid based on the two-phase model with an inclined magnetic field. The Buongiorno model-based analysis of an incompressible Walter's B fluid with the involvement of a rotatory cone was demonstrated by Gangadhar et al. [14].

The boundary layer flows produced by stretchable surfaces have numerous realistic utilizations including hot rolling, glass fiber manufacturing, metal extrusion, paper manufacturing, and metal spinning. There are different kinds of stretchable sheets namely linear, quadratic, exponential, power-law, and nonlinear. Yasir et al. [15] discuss the various features of the hybrid nanofluid flow near a stagnant point by assuming a porous stretchable surface. A scrutinization of the chemically reactive non-Newtonian fluid phenomenon near a stagnant point via a stretchable medium was conducted by Ishtiaq et al. [16]. The magnetized hybrid nanofluid flow mechanism produced by a stretchable cylinder was contemplated by Yasir et al. [17]. With the participation of numerous physical effects, Nadeem et al. [18] inspect the magnetized three-dimensional flow problem generated by a stretchable slander surface in a second-grade fluid having nanoparticles. Gangadhar et al. [19] assumed a stretchable cylinder to scrutinize the flow and heat transport mechanisms of a hybrid nanofluid influenced by heat absorption/generation. An exploration of the time-independent radiative flow mechanism through a stretchable surface was conducted by Nadeem et al. [20]. The magnetized flow phenomenon with the catalytic effects generated through a stretchable sheet in a nanofluid was demonstrated by Khan et al. [21].

The fluid flows with slip effects have many realistic implementations including refrigeration equipment, polymer solutions, mimicking biological water channels, cleaning of internal cavities, etc. Various researchers devoted their attention to exploring the significance of the partial slips on the different flows of fluid. Gangadhar et al. [22] worked on a water-based hybrid nanofluid to scrutinize the three-dimensional flow mechanism with the slip effects near a stagnant point. The consequence of the thermal slip on the steady flow mechanism of a fluid incorporating nanoparticles was examined by Wang et al. [23]. The significance of the partial slips on an incompressible radiative flow of Sutterby fluid subject to a stretchable medium was demonstrated by Sajid et al. [24]. An exploration of the hydromagnetic flow behavior of a hybrid nanofluid influenced by irregular slip impacts was conducted by Khan et al. [25]. More studies on the non-Newtonian fluids flow with slip effects can be found in Refs. [[26], [27], [28], [29], [30], [31]].

The motive of the current study is the time-independent three-dimensional flow analysis of a non-Newtonian Sutterby fluid subject to variable physical characteristics. This study has the novelty of the comparative analysis of Sutterby fluid flow on both exponential and nonlinear biaxially stretchable sheets. The impacts of the variable magnetic field, partial velocity slips, and variable heat sink/source are incorporated into the flow phenomenon. Moreover, the two-phase Buongiorno model is implemented to analyze the heat and mass transfer. The non-similar variables transform the constituting equations into a setup of dimensionless equations. In both cases of exponential and nonlinear surfaces, the concentration, temperature, and velocity distributions relative to the different physical parameters are physically visualized through graphs.

## 2 Mathematical description of the flow problem

**Fig. 1:**
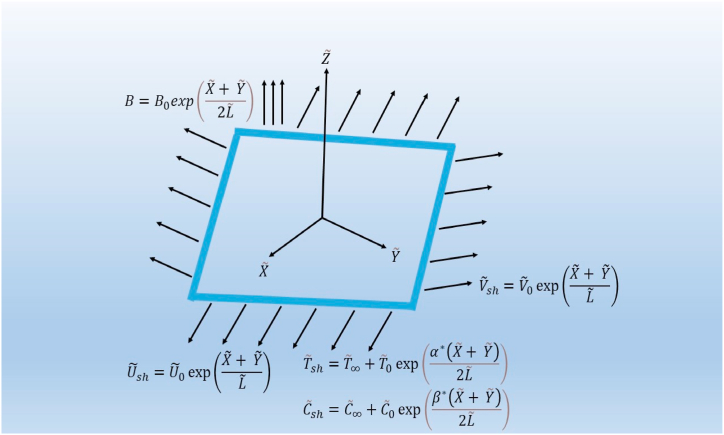
Schematic of exponential stretching sheet.

**Fig. 2:**
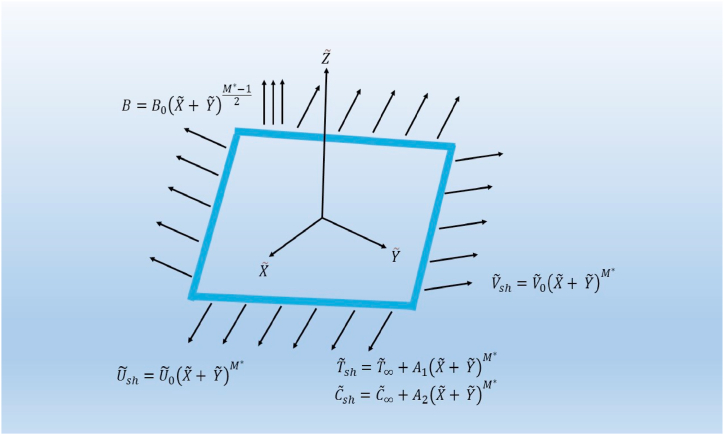
Schematic of exponential stretching sheet.

We consider two biaxial stretchable sheets (exponential and nonlinear) to investigate the boundary layer flow of a Sutterby nanofluid in three dimensions. In two lateral directions, the sheets are considered to be stretched. The physical setup are illustrated in Fig. 1 and Fig. 2, where the stretching sheets are situated at $\tilde{Z}=0$ and the direction of the $\tilde{Z}$-axis is normal to the $\tilde{X}\tilde{Y}$ plane, using a system of Cartesian coordinates. The region of the fluid flow is limited to $\tilde{Z}\geq 0$. The exponential sheet is stretching in the $\tilde{X}$ and $\tilde{Y}$ directions with velocities ${\tilde{U}}_{sh}={\tilde{U}}_{0}\;\exp\left(\frac{\tilde{X}+\tilde{Y}}{\tilde{L}}\right)$ and ${\tilde{V}}_{sh}={\tilde{V}}_{0}\;\exp\left(\frac{\tilde{X}+\tilde{Y}}{\tilde{L}}\right)$ respectively. Similarly, the stretchable velocities of the nonlinear sheet are ${\tilde{U}}_{sh}={\tilde{U}}_{0}{\left(\tilde{X}+\tilde{Y}\right)}^{M^{*}}$ and ${\tilde{V}}_{sh}={\tilde{V}}_{0}{\left(\tilde{X}+\tilde{Y}\right)}^{M^{*}}$ in the $\tilde{X}$ and $\tilde{Y}$ directions respectively. The exponential and nonlinear sheets are kept at temperatures ${\tilde{T}}_{sh}={\tilde{T}}_{\infty }+{\tilde{T}}_{0}\;\exp\left(\frac{\alpha^{*}\left(\tilde{X}+\tilde{Y}\right)}{2\tilde{L}}\right)$ and ${\tilde{T}}_{sh}={\tilde{T}}_{\infty }+A_{1}{\left(\tilde{X}+\tilde{Y}\right)}^{M^{*}}$ respectively while the concentrations of both sheets are taken as ${\tilde{C}}_{sh}={\tilde{C}}_{\infty }+{\tilde{C}}_{0}\;\exp\left(\frac{\beta^{*}\left(\tilde{X}+\tilde{Y}\right)}{2\tilde{L}}\right)$ and ${\tilde{C}}_{sh}={\tilde{C}}_{\infty }+A_{2}{\left(\tilde{X}+\tilde{Y}\right)}^{M^{*}}$ respectively. Variable magnetic fields $B=B_{0}\;\exp\left(\frac{\tilde{X}+\tilde{Y}}{2\tilde{L}}\right)$ and $B=B_{0}{\left(\tilde{X}+\tilde{Y}\right)}^{\frac{M^{*}-1}{2}}$ are implemented towards the $\tilde{Z}$ directions of the exponential and nonlinear sheets respectively. The effects of variable heat source/sink $Q=Q_{0}\;\exp\left(\frac{\tilde{X}+\tilde{Y}}{\tilde{L}}\right)$ and $Q=Q_{0}{\left(\tilde{X}+\tilde{Y}\right)}^{M^{*}-1}$ are incorporated in the heat transfer mechanism subject to exponential and nonlinear sheets respectively. Moreover, the Brownian motion and thermophoresis effects through the Buongiorno model and velocity slip impact are included in the steady flow problem.

For the considered Sutterby nanofluid, the constitutive equation has the following form of the Cauchy stress tensor [24].

(1) T=−pI+S

In Eq. (1), the extra stress tensor S has the following expressions [24]

(2) $$S=\mu_{0}{\left(\frac{\mathrm{arcsinh}\left(\beta \dot{\Gamma }\right)}{\beta \dot{\Gamma }}\right)}^{N}A$$

The power-law index N exhibit the shear thinning behavior of Sutterby fluid for N<0 and presents the shear thickening behavior for N>0.

After using second-order expansion $\sinh^{-1}\left(\beta \dot{\Gamma }\right)=\beta \dot{\Gamma }-\frac{\beta^{3}{\dot{\Gamma }}^{3}}{6}$, we have [24]

(3) $$S=\mu_{0}{\left(1-\frac{\beta^{2}{\dot{\Gamma }}^{2}}{6}\right)}^{N}A$$

here $\dot{\Gamma }$ and A has the following expressions [24]

(4) Γ˙=(12trA2)12,A=∇V+(∇V)t

For the concerned Sutterby nanofluid, the velocity field is taken as $V=\left(\tilde{U},\tilde{V},\tilde{W}\right)=\left(\tilde{U}\left(\tilde{X},\tilde{Y},\tilde{Z}\right),\tilde{V}\left(\tilde{X},\tilde{Y},\tilde{Z}\right),\tilde{W}\left(\tilde{X},\tilde{Y},\tilde{Z}\right)\right)$. So, Eq. (2) has the following components after using Eq. (3)

$$S_{\tilde{X}\tilde{X}}=2\mu_{0}{\left(1-\frac{\beta^{2}{\dot{\Gamma }}^{2}}{6}\right)}^{N}{\tilde{U}}_{\tilde{X}}$$

$$S_{\tilde{Y}\tilde{Y}}=2\mu_{0}{\left(1-\frac{\beta^{2}{\dot{\Gamma }}^{2}}{6}\right)}^{N}{\tilde{V}}_{\tilde{Y}}$$

$$S_{\tilde{Z}\tilde{Z}}=2\mu_{0}{\left(1-\frac{\beta^{2}{\dot{\Gamma }}^{2}}{6}\right)}^{N}{\tilde{W}}_{\tilde{Z}}$$

$$S_{\tilde{X}\tilde{Y}}=S_{\tilde{Y}\tilde{X}}=\mu_{0}{\left(1-\frac{\beta^{2}{\dot{\Gamma }}^{2}}{6}\right)}^{N}\left({\tilde{V}}_{\tilde{X}}+{\tilde{U}}_{\tilde{Y}}\right)$$

$$S_{\tilde{X}\tilde{Z}}=S_{\tilde{Z}\tilde{X}}=\mu_{0}{\left(1-\frac{\beta^{2}{\dot{\Gamma }}^{2}}{6}\right)}^{N}\left({\tilde{W}}_{\tilde{X}}+{\tilde{U}}_{\tilde{Z}}\right)$$

(5) $$S_{\tilde{Y}\tilde{Z}}=S_{\tilde{Z}\tilde{Y}}=\mu_{0}{\left(1-\frac{\beta^{2}{\dot{\Gamma }}^{2}}{6}\right)}^{N}\left({\tilde{V}}_{\tilde{Z}}+{\tilde{W}}_{\tilde{Y}}\right)$$

where

(6) $$\dot{\Gamma }={\left(\frac{1}{2{\left({\tilde{W}}_{\tilde{Z}}\right)}^{2}+2{\left({\tilde{V}}_{\tilde{Y}}\right)}^{2}+2{\left({\tilde{U}}_{\tilde{X}}\right)}^{2}+{\left({\tilde{W}}_{\tilde{X}}+{\tilde{U}}_{\tilde{Z}}\right)}^{2}+{\left({\tilde{V}}_{\tilde{X}}+{\tilde{U}}_{\tilde{Y}}\right)}^{2}+{\left({\tilde{V}}_{\tilde{Z}}+{\tilde{W}}_{\tilde{Y}}\right)}^{2}}\right)}^{-0.5}$$

in view of Eqs. (5), (6) and after using Boussinesq's approximation, the considered flow problem has the following governing equations [24,32,33].

(7) $${\tilde{U}}_{\tilde{X}}+{\tilde{V}}_{\tilde{Y}}+{\tilde{W}}_{\tilde{Z}}=0\text{,}$$

(8) $$\rho \left(\tilde{U}{\tilde{U}}_{\tilde{X}}+\tilde{V}{\tilde{U}}_{\tilde{Y}}+\tilde{W}{\tilde{U}}_{\tilde{Z}}\right)={\mu_{0}\left({\tilde{U}}_{\tilde{Z}}+\frac{N\beta^{2}}{6}{\left({\tilde{U}}_{\tilde{Z}}\right)}^{3}\right)}_{\tilde{Z}}-\sigma B^{2}\tilde{U}\text{,}$$

(9) $$\rho \left(\tilde{U}{\tilde{V}}_{\tilde{X}}+\tilde{V}{\tilde{V}}_{\tilde{Y}}+\tilde{W}{\tilde{V}}_{\tilde{Z}}\right)={\mu_{0}\left({\tilde{V}}_{\tilde{Z}}+\frac{N\beta^{2}}{6}{\left({\tilde{V}}_{\tilde{Z}}\right)}^{3}\right)}_{\tilde{Z}}-\sigma B^{2}\tilde{V}\text{,}$$

(10) $$\tilde{U}{\tilde{T}}_{\tilde{X}}+\tilde{V}{\tilde{T}}_{\tilde{Y}}+\tilde{W}{\tilde{T}}_{\tilde{Z}}=\frac{k}{\rho c_{p}}{\tilde{T}}_{\tilde{Z}\tilde{Z}}+\tau \left(D_{B}{\tilde{C}}_{\tilde{Z}}{\tilde{T}}_{\tilde{Z}}+\frac{D_{T}}{{\tilde{T}}_{\infty }}{\left({\tilde{T}}_{\tilde{Z}}\right)}^{2}\right)+\frac{Q}{\rho c_{p}}\left(\tilde{T}-{\tilde{T}}_{\infty }\right)\text{,}$$

(11) $$\tilde{U}{\tilde{C}}_{\tilde{X}}+\tilde{V}{\tilde{C}}_{\tilde{Y}}+\tilde{W}{\tilde{C}}_{\tilde{Z}}=D_{B}{\tilde{C}}_{\tilde{Z}\tilde{Z}}+\frac{D_{T}}{{\tilde{T}}_{\infty }}{\tilde{T}}_{\tilde{Z}\tilde{Z}}\text{,}$$

for an exponential sheet, the suitable boundary conditions are [33].

$$\tilde{U}={\tilde{U}}_{sh}\left(\tilde{X},\tilde{Y}\right)+N_{1}^{*}\left(\tilde{X},\tilde{Y}\right){\tilde{U}}_{\tilde{Z}},\tilde{V}={\tilde{V}}_{sh}\left(\tilde{X},\tilde{Y}\right)+N_{2}^{*}\left(\tilde{X},\tilde{Y}\right){\tilde{V}}_{\tilde{Z}},\tilde{W}=0\text{,}$$

$$\tilde{T}={\tilde{T}}_{sh},\tilde{C}={\tilde{C}}_{sh}\;at\;\tilde{Z}=0\text{,}$$

(12) $$\tilde{C}\to {\tilde{C}}_{\infty },\tilde{U}\to 0,\tilde{T}\to {\tilde{T}}_{\infty },\tilde{V}\to 0as\;\tilde{Z}\to \infty \text{.}$$

where $N_{1}^{*}\left(\tilde{X},\tilde{Y}\right)=N_{1}\;\exp\left(\frac{-\left(\tilde{X}+\tilde{Y}\right)}{2\tilde{L}}\right)$ and $N_{2}^{*}\left(\tilde{X},\tilde{Y}\right)=N_{2}\;\exp\left(\frac{-\left(\tilde{X}+\tilde{Y}\right)}{2\tilde{L}}\right)$.

For nonlinear sheet, the suitable boundary conditions are [[34], [35]].

$$\tilde{U}={\tilde{U}}_{sh}\left(\tilde{X},\tilde{Y}\right)+N_{1}^{*}\left(\tilde{X},\tilde{Y}\right){\tilde{U}}_{\tilde{Z}},\tilde{V}={\tilde{V}}_{sh}\left(\tilde{X},\tilde{Y}\right)+N_{2}^{*}\left(\tilde{X},\tilde{Y}\right){\tilde{V}}_{\tilde{Z}},\tilde{W}=0\text{,}$$

$$\tilde{T}={\tilde{T}}_{sh},\tilde{C}={\tilde{C}}_{sh}\;at\;\tilde{Z}=0\text{,}$$

(13) $$\tilde{U}\to 0,\tilde{T}\to {\tilde{T}}_{\infty },\tilde{V}\to 0,\tilde{C}\to {\tilde{C}}_{\infty }\;as\;\tilde{Z}\to \infty \text{.}$$

$$where\;N_{1}^{*}\left(\tilde{X},\tilde{Y}\right)=N_{1}{\left(\tilde{X}+\tilde{Y}\right)}^{\frac{1-M^{*}}{2}}\;and\;N_{2}^{*}\left(\tilde{X},\tilde{Y}\right)=N_{2}{\left(\tilde{X}+\tilde{Y}\right)}^{\frac{1-M^{*}}{2}}$$

Now the non-similar variables for an exponential sheet are defined as follows [33].

$$\tilde{U}={\tilde{U}}_{0}\;\exp\left(\frac{\tilde{X}+\tilde{Y}}{\tilde{L}}\right)\frac{dF}{d\zeta },\tilde{V}={\tilde{U}}_{0}\;\exp\left(\frac{\tilde{X}+\tilde{Y}}{\tilde{L}}\right)\frac{dG}{d\zeta }\text{,}$$

$$\tilde{W}=-{\left(\frac{\upsilon {\tilde{U}}_{0}}{2\tilde{L}}\right)}^{\frac{1}{2}}\exp\left(\frac{\tilde{X}+\tilde{Y}}{2\tilde{L}}\right)\left(F\left(\zeta \right)+\zeta \frac{dF}{d\zeta }+G\left(\zeta \right)+\zeta \frac{dG}{d\zeta }\right)\text{,}$$

(14) $$\tilde{T}={\tilde{T}}_{\infty }+{\tilde{T}}_{0}\;\exp\left(\frac{\alpha^{*}\left(\tilde{X}+\tilde{Y}\right)}{2\tilde{L}}\right)ϴ\left(\zeta \right),\tilde{C}={\tilde{C}}_{\infty }+{\tilde{C}}_{0}\;\exp\left(\frac{\beta^{*}\left(\tilde{X}+\tilde{Y}\right)}{2\tilde{L}}\right)\Phi \left(\zeta \right),\zeta ={\left(\frac{{\tilde{U}}_{0}}{2\upsilon \tilde{L}}\right)}^{\frac{1}{2}}\exp\left(\frac{\tilde{X}+\tilde{Y}}{2\tilde{L}}\right)\tilde{Z}\text{.}$$

For the nonlinear sheet, the non-similar variables are defined as follows [[34], [35]].

$$\tilde{U}={\tilde{U}}_{0}{\left(\tilde{X}+\tilde{Y}\right)}^{M^{*}}\frac{dF}{d\zeta },\tilde{V}={\tilde{U}}_{0}{\left(\tilde{X}+\tilde{Y}\right)}^{M^{*}}\frac{dG}{d\zeta }\text{,}$$

$$\tilde{W}=-{\left(\upsilon {\tilde{U}}_{0}\right)}^{\frac{1}{2}}{\left(\tilde{X}+\tilde{Y}\right)}^{\frac{M^{*}-1}{2}}\left(\frac{M^{*}-1}{2}\zeta \left(\frac{dF}{d\zeta }+\frac{dG}{d\zeta }\right)+\frac{M^{*}+1}{2}\left(F\left(\zeta \right)++G\left(\zeta \right)\right)\right)\text{,}$$

(15) $$\tilde{T}={\tilde{T}}_{\infty }+\left({\tilde{T}}_{sh}-{\tilde{T}}_{\infty }\right)ϴ\left(\zeta \right),\tilde{C}={\tilde{C}}_{\infty }+\left({\tilde{C}}_{sh}-{\tilde{C}}_{\infty }\right)\Phi \left(\zeta \right),\zeta ={\left(\frac{{\tilde{U}}_{0}}{\upsilon }\right)}^{\frac{1}{2}}{\left(\tilde{X}+\tilde{Y}\right)}^{\frac{M^{*}-1}{2}}\tilde{Z}\text{.}$$

after using Eq. (14), the setup of Eqs. (8), (9), (10), (11))) converts to the nonlinear equations for an exponential sheet as follows

(16) $$\frac{d^{3}F}{d\zeta^{3}}-2\frac{dF}{d\zeta }\left(\frac{dF}{d\zeta }+\frac{dG}{d\zeta }\right)+\frac{d^{2}F}{d\zeta^{2}}\left(F\left(\zeta \right)+G\left(\zeta \right)\right)+\frac{N}{4}DeRe{\left(\frac{d^{2}F}{d\zeta^{2}}\right)}^{2}\frac{d^{3}F}{d\zeta^{3}}-M\frac{dF}{d\zeta }=0\text{,}$$

(17) $$\frac{d^{3}G}{d\zeta^{3}}-2\frac{dG}{d\zeta }\left(\frac{dF}{d\zeta }+\frac{dG}{d\zeta }\right)+\frac{d^{2}G}{d\zeta^{2}}\left(F\left(\zeta \right)+G\left(\zeta \right)\right)+\frac{N}{4}DeRe{\left(\frac{d^{2}G}{d\zeta^{2}}\right)}^{2}\frac{d^{3}G}{d\zeta^{3}}-M\frac{dG}{d\zeta }=0\text{,}$$

(18) $$\frac{1}{\mathrm{Pr}}\frac{d^{2}ϴ}{d\zeta^{2}}-\alpha^{*}ϴ\left(\zeta \right)\left(\frac{dF}{d\zeta }+\frac{dG}{d\zeta }\right)+\frac{dϴ}{d\zeta }\left(F\left(\zeta \right)+G\left(\zeta \right)\right)+Nb\frac{dϴ}{d\zeta }\frac{d\Phi }{d\zeta }+Nt{\left(\frac{dϴ}{d\zeta }\right)}^{2}+Q^{*}ϴ\left(\zeta \right)=0\text{,}$$

(19) $$\frac{d^{2}\Phi }{d\zeta^{2}}+\frac{Nt}{Nb}\frac{d^{2}ϴ}{d\zeta^{2}}-Sc\left(\beta^{*}\Phi \left(\zeta \right)\left(\frac{dF}{d\zeta }+\frac{dG}{d\zeta }\right)-\frac{d\Phi }{d\zeta }\left(F\left(\zeta \right)+G\left(\zeta \right)\right)\right)=0\text{,}$$

for the nonlinear sheet, Eqs. (8), (9), (10), (11) transform to the following equations

(20) $$\frac{d^{3}F}{d\zeta^{3}}-M^{*}\frac{dF}{d\zeta }\left(\frac{dF}{d\zeta }+\frac{dG}{d\zeta }\right)+\left(\frac{M^{*}+1}{2}\right)\frac{d^{2}F}{d\zeta^{2}}\left(F\left(\zeta \right)+G\left(\zeta \right)\right)+\frac{N}{2}DeRe{\left(\frac{d^{2}F}{d\zeta^{2}}\right)}^{2}\frac{d^{3}F}{d\zeta^{3}}-M\frac{dF}{d\zeta }=0\text{,}$$

(21) $$\frac{d^{3}G}{d\zeta^{3}}-M^{*}\frac{dG}{d\zeta }\left(\frac{dF}{d\zeta }+\frac{dG}{d\zeta }\right)+\left(\frac{M^{*}+1}{2}\right)\frac{d^{2}G}{d\zeta^{2}}\left(F\left(\zeta \right)+G\left(\zeta \right)\right)+\frac{N}{2}DeRe{\left(\frac{d^{2}G}{d\zeta^{2}}\right)}^{2}\frac{d^{3}G}{d\zeta^{3}}-M\frac{dG}{d\zeta }=0$$

(22) $$\frac{1}{\mathrm{Pr}}\frac{d^{2}ϴ}{d\zeta^{2}}-M^{*}ϴ\left(\zeta \right)\left(\frac{dF}{d\zeta }+\frac{dG}{d\zeta }\right)+\left(\frac{M^{*}+1}{2}\right)\frac{dϴ}{d\zeta }\left(F\left(\zeta \right)+G\left(\zeta \right)\right)+Nb\frac{dϴ}{d\zeta }\frac{d\Phi }{d\zeta }+Nt{\left(\frac{dϴ}{d\zeta }\right)}^{2}+Q^{*}ϴ\left(\zeta \right)=0$$

(23) $$\frac{d^{2}\Phi }{d\zeta^{2}}+\frac{Nt}{Nb}\frac{d^{2}ϴ}{d\zeta^{2}}-Sc\left(M^{*}\Phi \left(\zeta \right)\left(\frac{dF}{d\zeta }+\frac{dG}{d\zeta }\right)-\left(\frac{M^{*}+1}{2}\right)\frac{d\Phi }{d\zeta }\left(F\left(\zeta \right)+G\left(\zeta \right)\right)\right)=0\text{,}$$

The above equations have the following transformed boundary conditions

$$F\left(0\right)=0,\frac{dF\left(0\right)}{d\zeta }=1+\xi_{1}\frac{d^{2}F\left(0\right)}{d\zeta^{2}},G\left(0\right)=0,\frac{dG\left(0\right)}{d\zeta }=\delta +\xi_{2}\frac{d^{2}G\left(0\right)}{d\zeta^{2}},ϴ\left(0\right)=1,\Phi \left(0\right)=1$$

(24) $$\frac{dF}{d\zeta }\to 0,ϴ\left(\zeta \right)\to 0,\frac{dG}{d\zeta }\to 0,\Phi \left(\zeta \right)\to 0\;as\;\zeta \to \infty \text{.}$$

The parameters involved in Eqs. (16), (17), (18), (19), (24) for exponential sheet are defined as follows

$$M=\frac{2\sigma {B_{0}}^{2}\tilde{L}}{\rho {\tilde{U}}_{0}},De={{\tilde{U}}_{0}}^{2}\beta^{2},Re=\frac{{{\tilde{U}}_{sh}}^{3}}{{{\tilde{U}}_{0}}^{2}\upsilon \tilde{L}},Q^{*}=\frac{2\tilde{L}Q}{{\tilde{U}}_{0}\rho c_{p}},Nb=\frac{\tau D_{B}\left({\tilde{C}}_{sh}-{\tilde{C}}_{\infty }\right)}{\upsilon }\text{,}$$

(25) $$\mathrm{Pr}=\frac{\upsilon }{\alpha },Nt=\frac{\tau D_{T}\left({\tilde{T}}_{sh}-{\tilde{T}}_{\infty }\right)}{\upsilon {\tilde{T}}_{\infty }},Sc={\left(\frac{D_{B}}{\upsilon }\right)}^{-1},\xi_{1}=N_{1}{\left(\frac{{\tilde{U}}_{0}}{2\upsilon \tilde{L}}\right)}^{\frac{1}{2}},\delta =\frac{{\tilde{V}}_{0}}{{\tilde{U}}_{0}}\;\xi_{2}=N_{2}{\left(\frac{{\tilde{U}}_{0}}{2\upsilon \tilde{L}}\right)}^{\frac{1}{2}}$$

The parameters involved in Eqs. (20), (21), (22), (23), (24) for the nonlinear sheet are defined as follows

$$De={{\tilde{U}}_{0}}^{2}\beta^{2},M=\frac{\sigma {B_{0}}^{2}}{\rho {\tilde{U}}_{0}},Re=\frac{{{\tilde{U}}_{sh}}^{3}{\left(\tilde{X}+\tilde{Y}\right)}^{-1}}{{{\tilde{U}}_{0}}^{2}\upsilon },Nt=\frac{\tau D_{T}\left({\tilde{T}}_{sh}-{\tilde{T}}_{\infty }\right)}{\upsilon {\tilde{T}}_{\infty }},\mathrm{Pr}=\frac{\upsilon }{\alpha }\text{,}$$

(26) $$Nb=\frac{\tau D_{B}\left({\tilde{C}}_{sh}-{\tilde{C}}_{\infty }\right)}{\upsilon },Q^{*}=\frac{Q_{0}}{{\tilde{U}}_{0}\rho c_{p}},Sc={\left(\frac{D_{B}}{\upsilon }\right)}^{-1},\xi_{1}=N_{1}{\left(\frac{{\tilde{U}}_{0}}{\upsilon }\right)}^{\frac{1}{2}},\delta =\frac{{\tilde{V}}_{0}}{{\tilde{U}}_{0}}\;\xi_{2}=N_{2}{\left(\frac{{\tilde{U}}_{0}}{\upsilon }\right)}^{\frac{1}{2}}\text{.}$$

## 3 Solution technique

To acquire the numerical solution of Eqs. (16), (17), (18), (19), (20), (21), (22), (23) subject to Eq. (24), we implement an effective bvp4c methodology in MATLAB. The bvp4c technique relies on the finite difference method. The collocation three-stage Lobatto IIIa formula is adopted in this technique. A $C^{1}$-continuous solution provided by the collocation polynomial is uniformly accurate to the fourth order in the interval of integration. The error control is dependent on the continuous solution's residual. In the bvp4c technique, the nonlinear system of higher-order equations is transformed into a setup of first-order equations. For an exponential sheet, Eqs. (16), (17), (18), (19) can be written as follows

(27) $$\frac{d^{3}F}{d\zeta^{3}}=\left(\frac{1}{1+\frac{N}{4}DeRe{\left(\frac{d^{2}F}{d\zeta^{2}}\right)}^{2}}\right)\left(2\frac{dF}{d\zeta }\left(\frac{dF}{d\zeta }+\frac{dG}{d\zeta }\right)-\frac{d^{2}F}{d\zeta^{2}}\left(F\left(\zeta \right)+G\left(\zeta \right)\right)+M\frac{dF}{d\zeta }\right)$$

(28) $$\frac{d^{3}G}{d\zeta^{3}}=\left(\frac{1}{1+\frac{N}{4}DeRe{\left(\frac{d^{2}G}{d\zeta^{2}}\right)}^{2}}\right)\left(2\frac{dG}{d\zeta }\left(\frac{dF}{d\zeta }+\frac{dG}{d\zeta }\right)-\frac{d^{2}G}{d\zeta^{2}}\left(F\left(\zeta \right)+G\left(\zeta \right)\right)+M\frac{dG}{d\zeta }\right)$$

(29) $$\frac{d^{2}ϴ}{d\zeta^{2}}=\mathrm{Pr}\left(\alpha^{*}ϴ\left(\zeta \right)\left(\frac{dF}{d\zeta }+\frac{dG}{d\zeta }\right)-\frac{dϴ}{d\zeta }\left(F\left(\zeta \right)+G\left(\zeta \right)\right)-Nb\frac{dϴ}{d\zeta }\frac{d\Phi }{d\zeta }-Nt{\left(\frac{dϴ}{d\zeta }\right)}^{2}-Q^{*}ϴ\left(\zeta \right)\right)$$

(30) $$\frac{d^{2}\Phi }{d\zeta^{2}}=Sc\left(\alpha^{*}\Phi \left(\zeta \right)\left(\frac{dF}{d\zeta }+\frac{dG}{d\zeta }\right)-\frac{d\Phi }{d\zeta }\left(F\left(\zeta \right)+G\left(\zeta \right)\right)-\frac{Nt}{Nb}\frac{d^{2}ϴ}{d\zeta^{2}}\right)$$

Now, we introduce new variables to transform the above Eqs. (27), (28), (29), (30) into the setup of first-order equations as follows

$$F\left(\zeta \right)=Y_{1},\frac{dF}{d\zeta }=Y_{2},\frac{d^{2}F}{d\zeta^{2}}=Y_{3},\frac{d^{3}F}{d\zeta^{3}}=Y_{3}^{\prime }\text{,}$$

$$G\left(\zeta \right)=Y_{4},\frac{dG}{d\zeta }=Y_{5},\frac{d^{2}G}{d\zeta^{2}}=Y_{6},\frac{d^{3}G}{d\zeta^{3}}=Y_{6}^{\prime }\text{,}$$

(31) $$ϴ\left(\zeta \right)=Y_{7},\frac{dϴ}{d\zeta }=Y_{8},\frac{d^{2}ϴ}{d\zeta^{2}}=Y_{8}^{\prime },\Phi \left(\zeta \right)=Y_{9},\frac{d\Phi }{d\zeta }=Y_{10},\frac{d^{2}\Phi }{d\zeta^{2}}=Y_{10}^{\prime }\text{.}$$

now, we have

$$Y_{2}=Y_{1}^{\prime }\text{,}$$

$$Y_{3}=Y_{2}^{\prime }\text{,}$$

(32) $$Y_{3}^{\prime }=\left(\frac{1}{1+\frac{N}{4}DeRe{\left(Y_{3}\right)}^{2}}\right)\left(2Y_{2}\left(Y_{2}+Y_{5}\right)-Y_{3}\left(Y_{1}+Y_{4}\right)+MY_{2}\right)\text{,}$$

$$Y_{5}=Y_{4}^{\prime }\text{,}$$

$$Y_{6}=Y_{5}^{\prime }\text{,}$$

(33) $$Y_{6}^{\prime }=\left(\frac{1}{1+\frac{N}{4}DeRe{\left(Y_{6}\right)}^{2}}\right)\left(2Y_{5}\left(Y_{2}+Y_{5}\right)-Y_{6}\left(Y_{1}+Y_{4}\right)+MY_{5}\right)\text{,}$$

$$Y_{8}={Y_{7}}^{\prime }$$

(34) $$Y_{8}^{\prime }=\mathrm{Pr}\left(\alpha^{*}Y_{7}\left(Y_{2}+Y_{5}\right)-Y_{8}\left(Y_{1}+Y_{4}\right)-NbY_{8}Y_{10}-Nt{\left(Y_{8}\right)}^{2}-Q^{*}Y_{7}\right)$$

$$Y_{10}={Y_{9}}^{\prime }$$

(35) $$Y_{10}^{\prime }=Sc\left(\alpha^{*}Y_{9}\left(Y_{2}+Y_{5}\right)-Y_{10}\left(Y_{1}+Y_{4}\right)-\frac{Nt}{Nb}Y_{8}^{\prime }\right)$$

The system of first-order equations for the nonlinear sheet is defined as follows

$$F\left(\zeta \right)=Z_{1},\frac{dF}{d\zeta }=Z_{2},\frac{d^{2}F}{d\zeta^{2}}=Z_{3},\frac{d^{3}F}{d\zeta^{3}}=Z_{3}^{\prime }\text{,}$$

$$G\left(\zeta \right)=Z_{4},\frac{dG}{d\zeta }=Z_{5},\frac{d^{2}G}{d\zeta^{2}}=Z_{6},\frac{d^{3}G}{d\zeta^{3}}=Z_{6}^{\prime }\text{,}$$

(36) $$ϴ\left(\zeta \right)=Z_{7},\frac{dϴ}{d\zeta }=Z_{8},\frac{d^{2}ϴ}{d\zeta^{2}}=Z_{8}^{\prime },\Phi \left(\zeta \right)=Z_{9},\frac{d\Phi }{d\zeta }=Z_{10},\frac{d^{2}\Phi }{d\zeta^{2}}=Z_{10}^{\prime }\text{.}$$

$$Z_{2}=Z_{1}^{\prime }\text{,}$$

$$Z_{3}=Z_{2}^{\prime }\text{,}$$

(37) $$Z_{3}^{\prime }=\left(\frac{1}{1+\frac{N}{2}DeRe{\left(Z_{3}\right)}^{2}}\right)\left(M^{*}Z_{2}\left(Z_{2}+Z_{5}\right)-\left(\frac{M^{*}+1}{2}\right)Z_{3}\left(Z_{1}+Z_{4}\right)+MZ_{2}\right)\text{,}$$

$$Z_{5}=Z_{4}^{\prime }\text{,}$$

$$Z_{6}=Z_{5}^{\prime }\text{,}$$

(38) $$Z_{6}^{\prime }=\left(\frac{1}{1+\frac{N}{2}DeRe{\left(Z_{6}\right)}^{2}}\right)\left(M^{*}Z_{5}\left(Z_{2}+Z_{5}\right)-\left(\frac{M^{*}+1}{2}\right)Z_{6}\left(Z_{1}+Z_{4}\right)+MZ_{5}\right)\text{,}$$

$$Z_{8}={Z_{7}}^{\prime }$$

(39) $$Z_{8}^{\prime }=\mathrm{Pr}\left(M^{*}Z_{7}\left(Z_{2}+Z_{5}\right)-\left(\frac{M^{*}+1}{2}\right)Z_{8}\left(Z_{1}+Z_{4}\right)-NbZ_{8}Z_{10}-Nt{\left(Z_{8}\right)}^{2}-Q^{*}Z_{7}\right)$$

$$Z_{10}={Z_{9}}^{\prime }$$

(40) $$Z_{10}^{\prime }=Sc\left(M^{*}Z_{9}\left(Z_{2}+Z_{5}\right)-\left(\frac{M^{*}+1}{2}\right)Z_{10}\left(Z_{1}+Z_{4}\right)-\frac{Nt}{Nb}Z_{8}^{\prime }\right)$$

by using new variables, the boundary conditions have the following expressions

$$Y_{0}\left(1\right)=0,Y_{0}\left(2\right)=1+\xi_{1}Y_{0}\left(3\right),Y_{0}\left(4\right)=0,Y_{0}\left(5\right)=\delta +\xi_{2}Y_{0}\left(6\right),Y_{0}\left(7\right)=1,Y_{0}\left(9\right)=1\text{,}$$

$$Y_{\inf}\left(2\right),Y_{\inf}\left(5\right),Y_{\inf}\left(7\right),Y_{\inf}\left(9\right)\text{.}$$

$$Z_{0}\left(1\right)=0,Z_{0}\left(2\right)=1+\xi_{1}Z_{0}\left(3\right),Z_{0}\left(4\right)=0,Z_{0}\left(5\right)=\delta +\xi_{2}Z_{0}\left(6\right),Z_{0}\left(7\right)=1,Z_{0}\left(9\right)=1\text{,}$$

(41) $$Z_{\inf}\left(2\right),Z_{\inf}\left(5\right),Z_{\inf}\left(7\right),Z_{\inf}\left(9\right)\text{.}$$

## 4 Physical quantities

The physical interesting quantities associated with the ongoing flow problem include skin friction coefficients, Sherwood number, and local Nusselt number. These quantities signify the transfer of heat, mass, and momentum respectively. For an exponential sheet, the mathematical expressions of the skin friction coefficient along $\tilde{X}$ and $\tilde{Y}$ directions, local Nusselt number, and Sherwood number are defined as follows [33]

(42) $${Cf}_{\tilde{X}}=\frac{2\tau_{\tilde{Z}\tilde{X}}}{\rho {{\tilde{U}}_{sh}}^{2}},{Cf}_{\tilde{Y}}=\frac{2\tau_{\tilde{Z}\tilde{Y}}}{\rho {{\tilde{U}}_{sh}}^{2}},{Nu}_{\tilde{X}}=\frac{\tilde{X}q_{w}}{k\left({\tilde{T}}_{sh}-{\tilde{T}}_{\infty }\right)},{Sh}_{\tilde{X}}=\frac{\tilde{X}q_{m}}{D_{B}\left({\tilde{C}}_{sh}-{\tilde{C}}_{\infty }\right)}$$

where

$$\tau_{\tilde{Z}\tilde{X}}={\mu_{0}\left({\tilde{U}}_{\tilde{Z}}+\frac{N\beta^{2}}{6}{\left({\tilde{U}}_{\tilde{Z}}\right)}^{3}\right)|}_{\tilde{Z}=0},\tau_{\tilde{Z}\tilde{Y}}={\mu_{0}\left({\tilde{V}}_{\tilde{Z}}+\frac{N\beta^{2}}{6}{\left({\tilde{V}}_{\tilde{Z}}\right)}^{3}\right)|}_{\tilde{Z}=0}\text{,}$$

(43) $$q_{w}={-k{\tilde{T}}_{\tilde{Z}}|}_{\tilde{Z}=0},q_{m}={-D_{B}{\tilde{C}}_{\tilde{Z}}|}_{\tilde{Z}=0}$$

after using Eq. (43) in (42), we get the following form

$${\left(\frac{{Re}_{\tilde{X}}}{2}\right)}^{0.5}{Cf}_{\tilde{X}}=\frac{d^{2}F\left(0\right)}{d\zeta^{2}}+\frac{N}{12}ReDe{\left(\frac{d^{2}F\left(0\right)}{d\zeta^{2}}\right)}^{3},{\left(\frac{{Re}_{\tilde{Y}}}{2}\right)}^{0.5}{Cf}_{\tilde{Y}}=\frac{d^{2}G\left(0\right)}{d\zeta^{2}}+\frac{N}{12}ReDe{\left(\frac{d^{2}G\left(0\right)}{d\zeta^{2}}\right)}^{3}$$

(44) $${\left(\frac{2}{{Re}_{\tilde{X}}}\right)}^{0.5}{Nu}_{\tilde{X}}=-\frac{dϴ\left(0\right)}{d\zeta },{\left(\frac{2}{{Re}_{\tilde{X}}}\right)}^{0.5}{Sh}_{\tilde{X}}=-\frac{d\Phi \left(0\right)}{d\zeta }\text{.}$$

where ${Re}_{\tilde{X}}=\frac{{\tilde{U}}_{sh}\tilde{L}}{\upsilon }$ and ${Re}_{\tilde{Y}}=\frac{{\tilde{V}}_{sh}\tilde{L}}{\upsilon }$.

For nonlinear sheet, the physical quantities have the following expressions [[34], [35]].

(45) $${Cf}_{\tilde{X}}=\frac{\tau_{\tilde{Z}\tilde{X}}}{\rho {{\tilde{U}}_{sh}}^{2}},{Cf}_{\tilde{Y}}=\frac{\tau_{\tilde{Z}\tilde{Y}}}{\rho {{\tilde{U}}_{sh}}^{2}},{Nu}_{\tilde{X}}=\frac{\left(\tilde{X}+\tilde{Y}\right)q_{w}}{k\left({\tilde{T}}_{sh}-{\tilde{T}}_{\infty }\right)},{Sh}_{\tilde{X}}=\frac{\left(\tilde{X}+\tilde{Y}\right)q_{m}}{D_{B}\left({\tilde{C}}_{sh}-{\tilde{C}}_{\infty }\right)}$$

where

$$\tau_{\tilde{Z}\tilde{X}}={\mu_{0}\left({\tilde{U}}_{\tilde{Z}}+\frac{N\beta^{2}}{6}{\left({\tilde{U}}_{\tilde{Z}}\right)}^{3}\right)|}_{\tilde{Z}=0},\tau_{\tilde{Z}\tilde{Y}}={\mu_{0}\left({\tilde{V}}_{\tilde{Z}}+\frac{N\beta^{2}}{6}{\left({\tilde{V}}_{\tilde{Z}}\right)}^{3}\right)|}_{\tilde{Z}=0}\text{,}$$

(46) $$q_{w}={-k{\tilde{T}}_{\tilde{Z}}|}_{\tilde{Z}=0},q_{m}={-D_{B}{\tilde{C}}_{\tilde{Z}}|}_{\tilde{Z}=0}$$

after using Eq. (46) in (45), we get the following form

$${\left({Re}_{\tilde{X}}\right)}^{0.5}{Cf}_{\tilde{X}}=\frac{d^{2}F\left(0\right)}{d\zeta^{2}}+\frac{N}{6}ReDe{\left(\frac{d^{2}F\left(0\right)}{d\zeta^{2}}\right)}^{3},{\left({Re}_{\tilde{Y}}\right)}^{0.5}{Cf}_{\tilde{Y}}=\frac{d^{2}G\left(0\right)}{d\zeta^{2}}+\frac{N}{6}ReDe{\left(\frac{d^{2}G\left(0\right)}{d\zeta^{2}}\right)}^{3}$$

(47) $${\left(\frac{1}{{Re}_{\tilde{X}}}\right)}^{0.5}{Nu}_{\tilde{X}}=-\frac{dϴ\left(0\right)}{d\zeta },{\left(\frac{1}{{Re}_{\tilde{X}}}\right)}^{0.5}{Sh}_{\tilde{X}}=-\frac{d\Phi \left(0\right)}{d\zeta }\text{.}$$

$$where\;{Re}_{\tilde{X}}=\frac{{\tilde{U}}_{sh}{\left(\tilde{X}+\tilde{Y}\right)}^{M^{*}+1}}{\upsilon }\;and\;{Re}_{\tilde{Y}}=\frac{{\tilde{V}}_{sh}{\left(\tilde{X}+\tilde{Y}\right)}^{M^{*}+1}}{\upsilon }$$

## 5 Outcomes and discussion

**Fig. 3:**
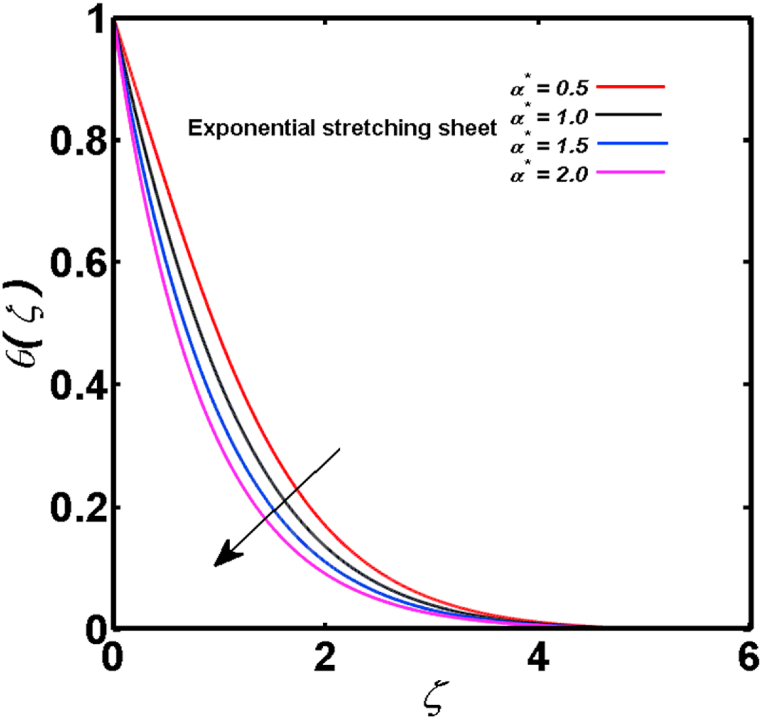
Curve of ϴ(ζ) regarding $\alpha^{*}$ parameter.

**Fig. 4:**
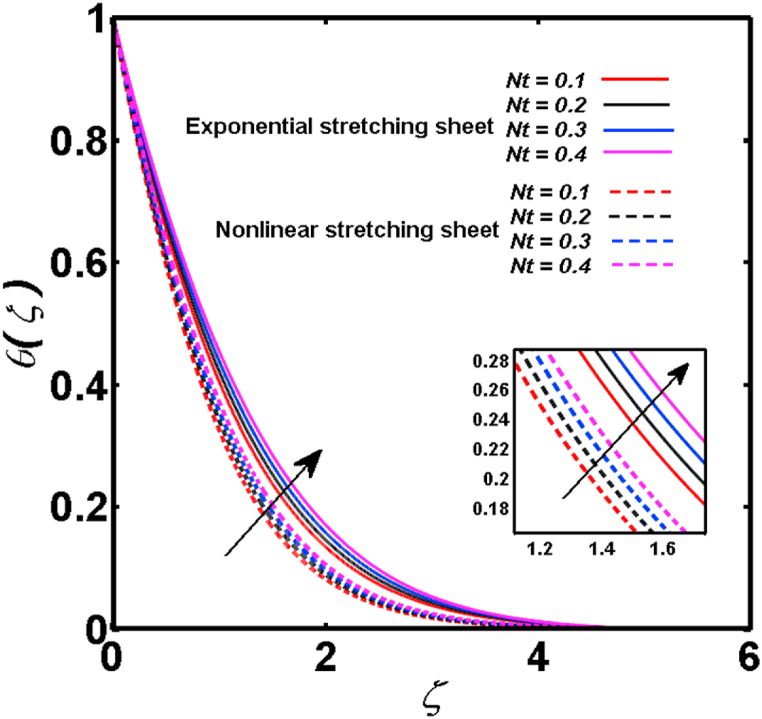
Curve of ϴ(ζ) regarding Nt parameter.

**Fig. 5:**
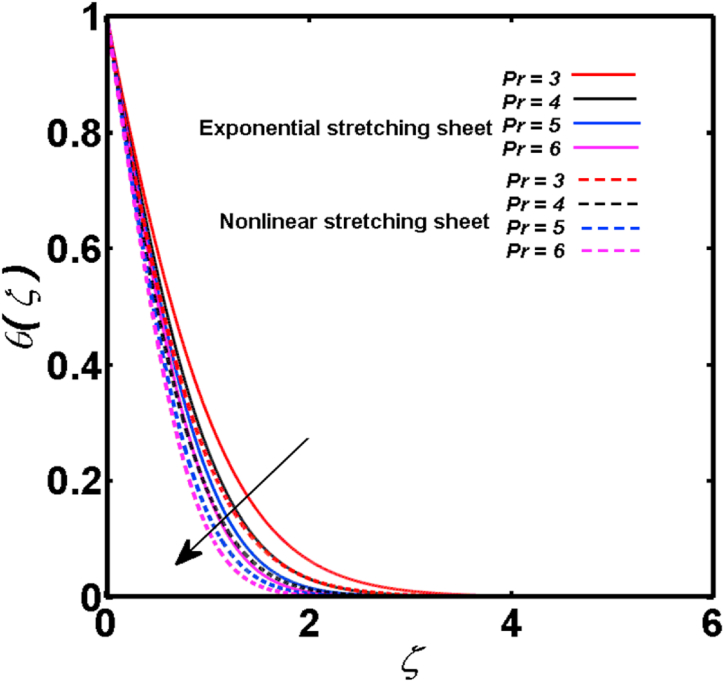
Curve of ϴ(ζ) regarding Pr parameter.

**Fig. 6:**
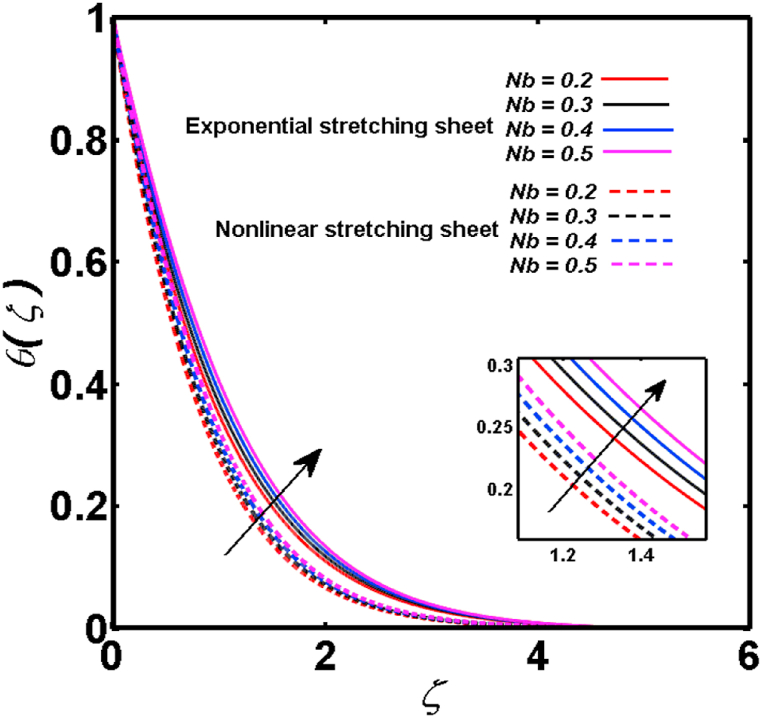
Curve of ϴ(ζ) regarding Nb parameter.

**Fig. 7:**
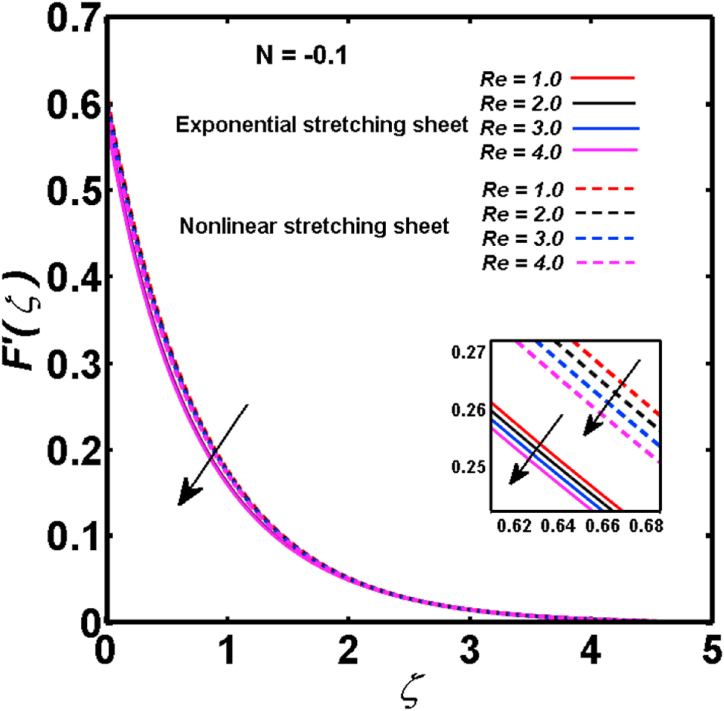
For shear thinning fluid, curve of $F^{\prime }\left(\zeta \right)$ regarding Re parameter.

**Fig. 8:**
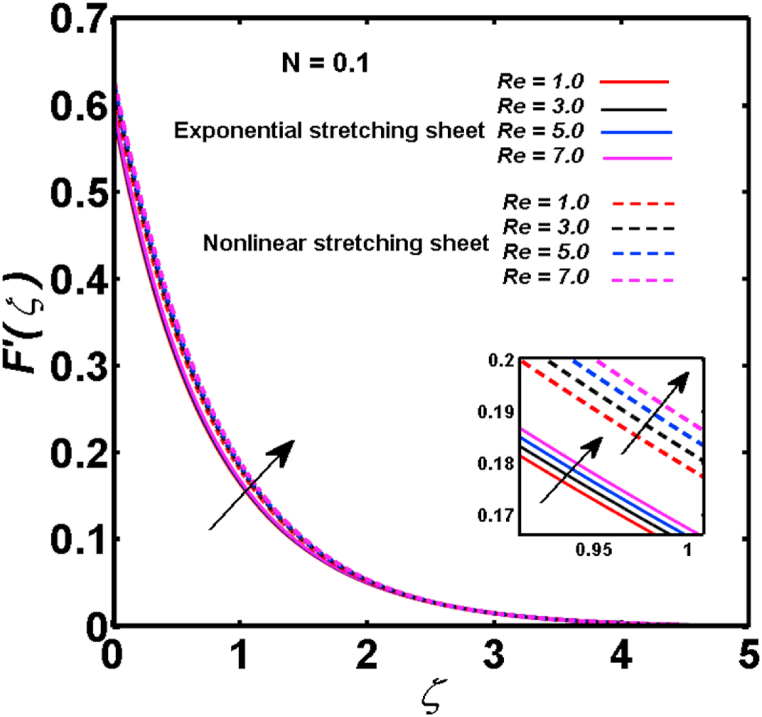
For shear thickening fluid, curve of $F^{\prime }\left(\zeta \right)$ regarding Re parameter.

**Fig. 9:**
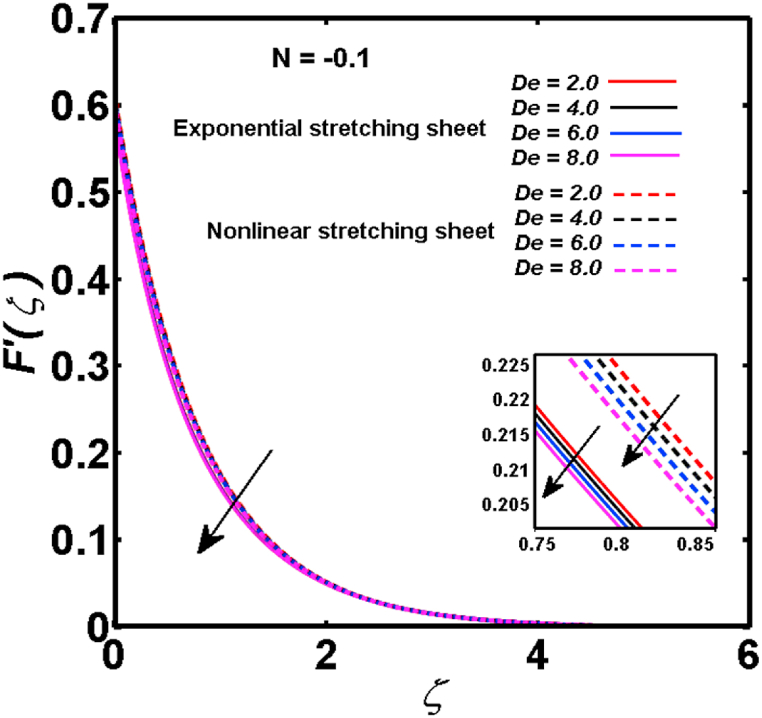
For shear thinning fluid, curve of $F^{\prime }\left(\zeta \right)$ regarding De parameter.

**Fig. 10:**
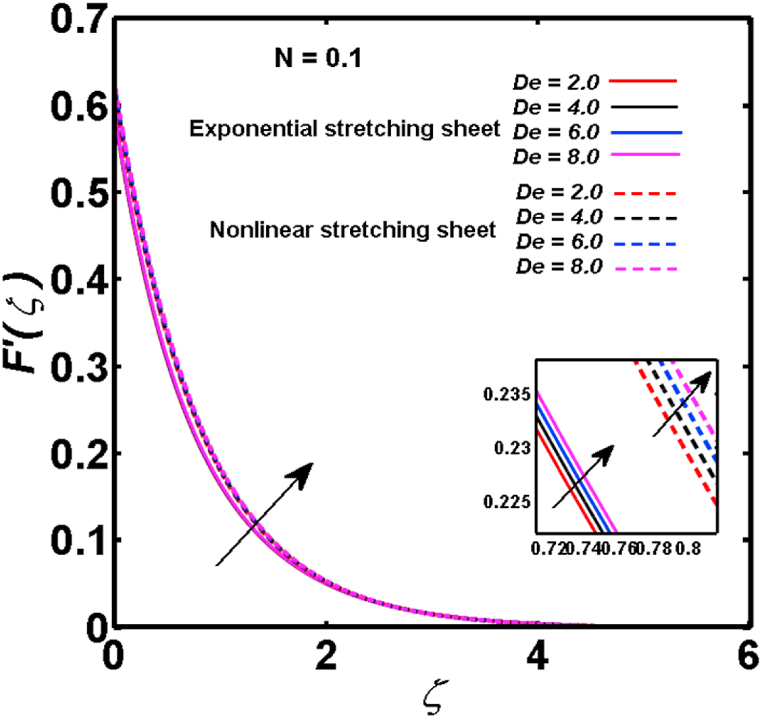
For shear thickening fluid, curve of $F^{\prime }\left(\zeta \right)$ regarding De parameter.

**Fig. 11:**
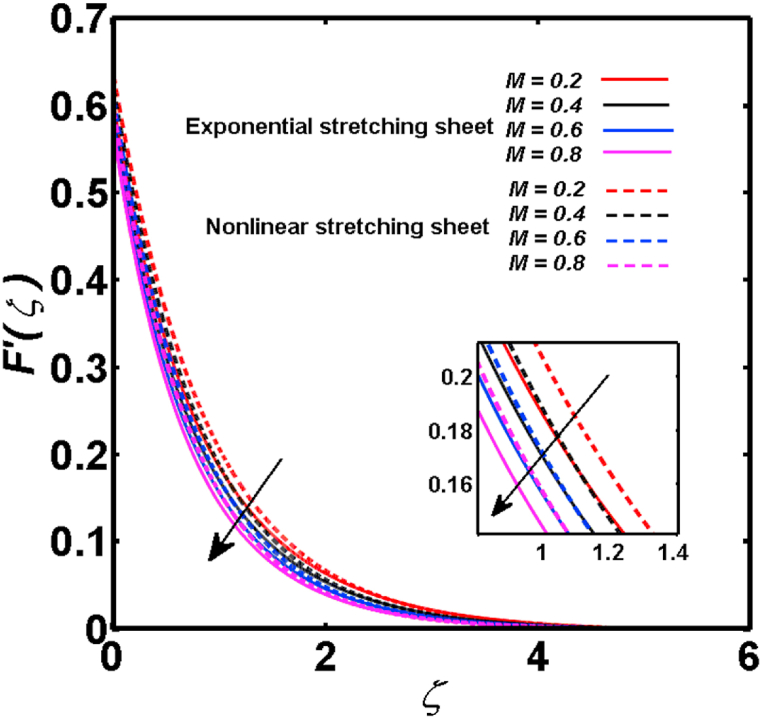
Curve of $F^{\prime }\left(\zeta \right)$ regarding M parameter.

**Fig. 12:**
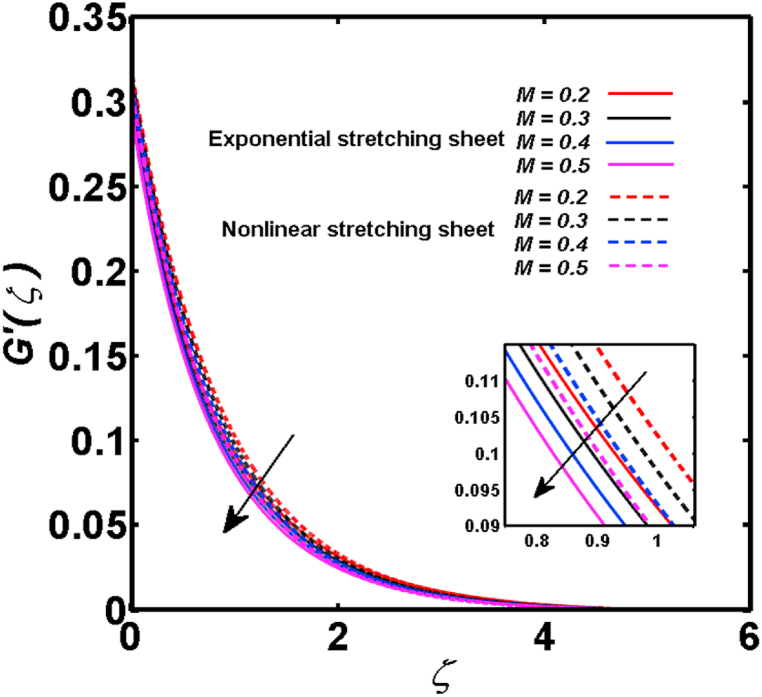
Curve of $G^{\prime }\left(\zeta \right)$ regarding M parameter.

**Fig. 13:**
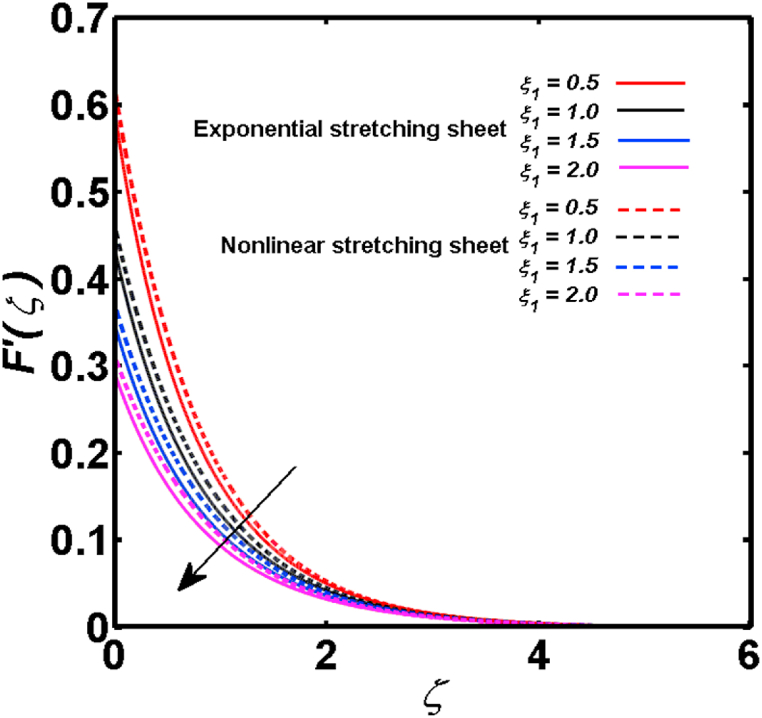
Curve of $F^{\prime }\left(\zeta \right)$ regarding $\xi_{1}$ parameter.

**Fig. 14:**
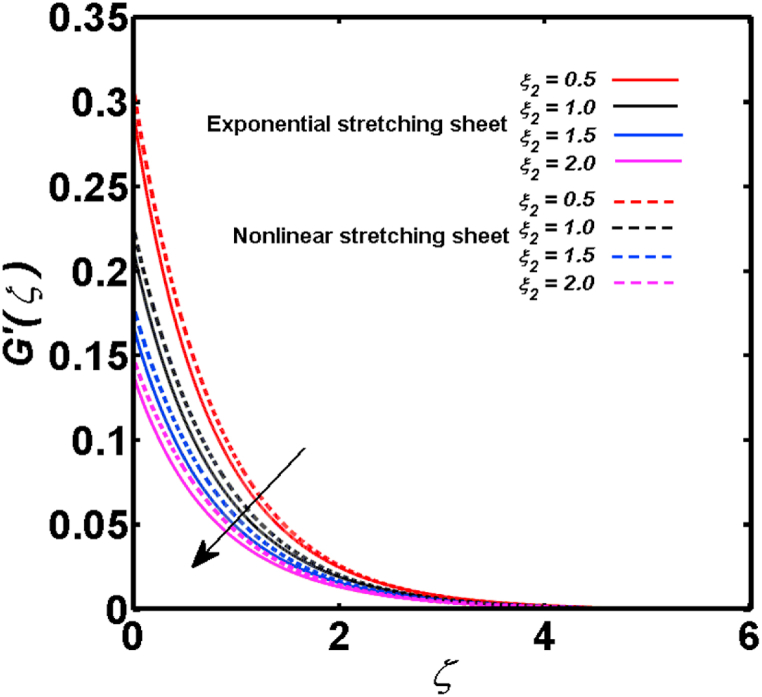
Curve of $G^{\prime }\left(\zeta \right)$ regarding $\xi_{2}$ parameter.

**Fig. 15:**
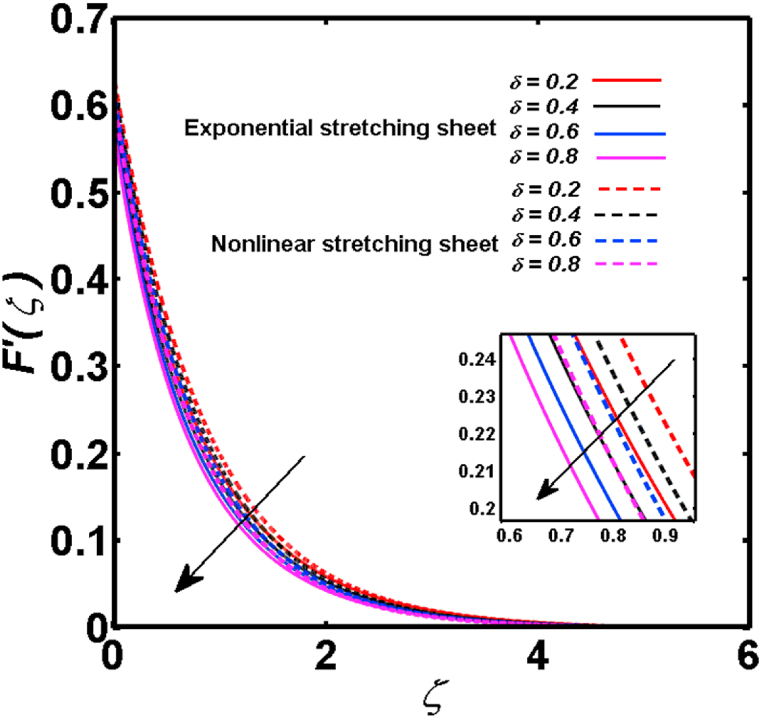
Curve of $F^{\prime }\left(\zeta \right)$ regarding δ parameter.

**Fig. 16:**
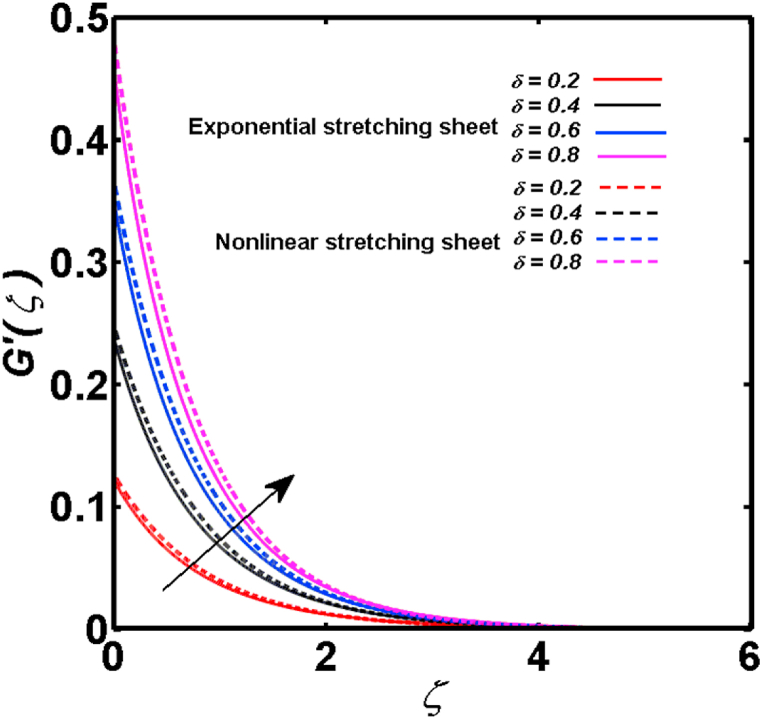
Curve of $G^{\prime }\left(\zeta \right)$ regarding δ parameter.

**Fig. 17:**
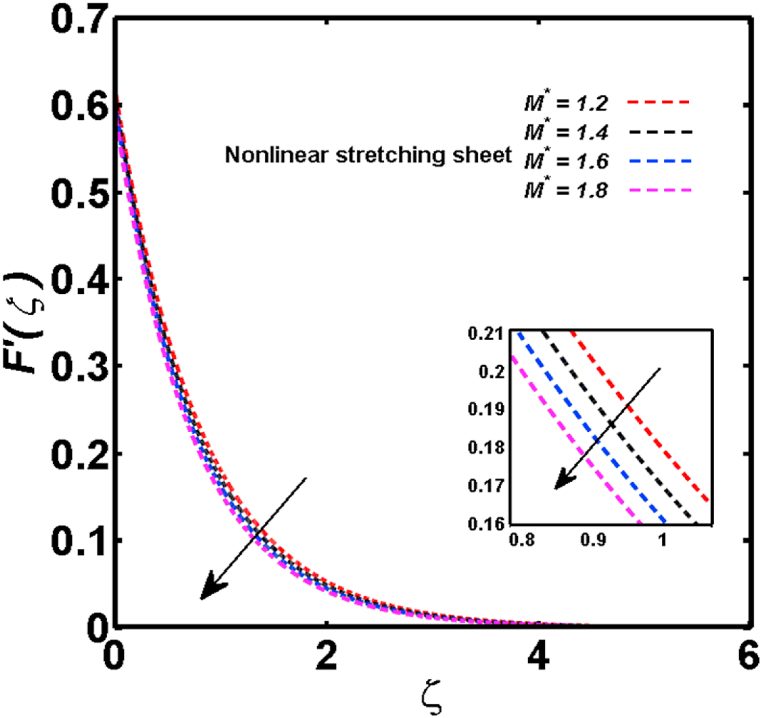
Curve of $F^{\prime }\left(\zeta \right)$ regarding $M^{*}$ parameter.

**Fig. 18:**
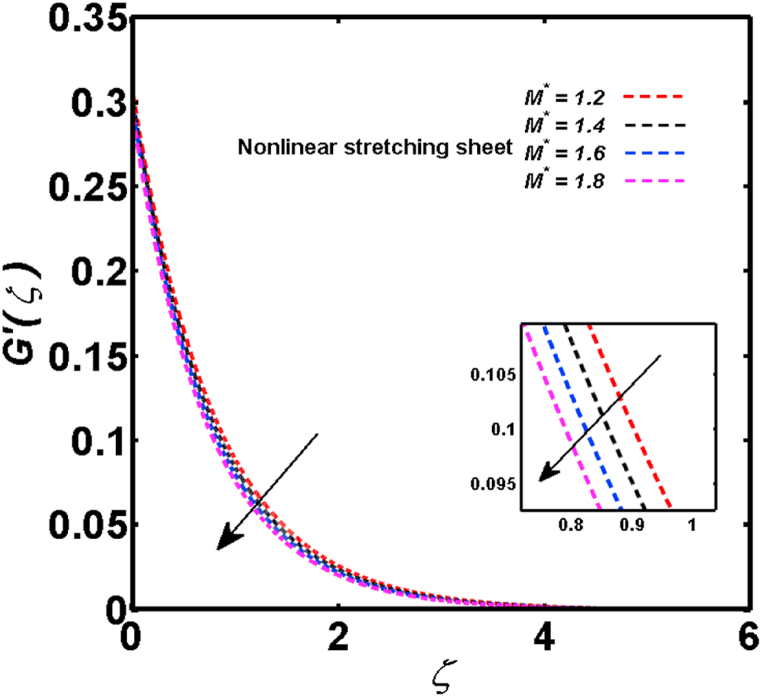
Curve of $G^{\prime }\left(\zeta \right)$ regarding $M^{*}$ parameter.

**Fig. 19:**
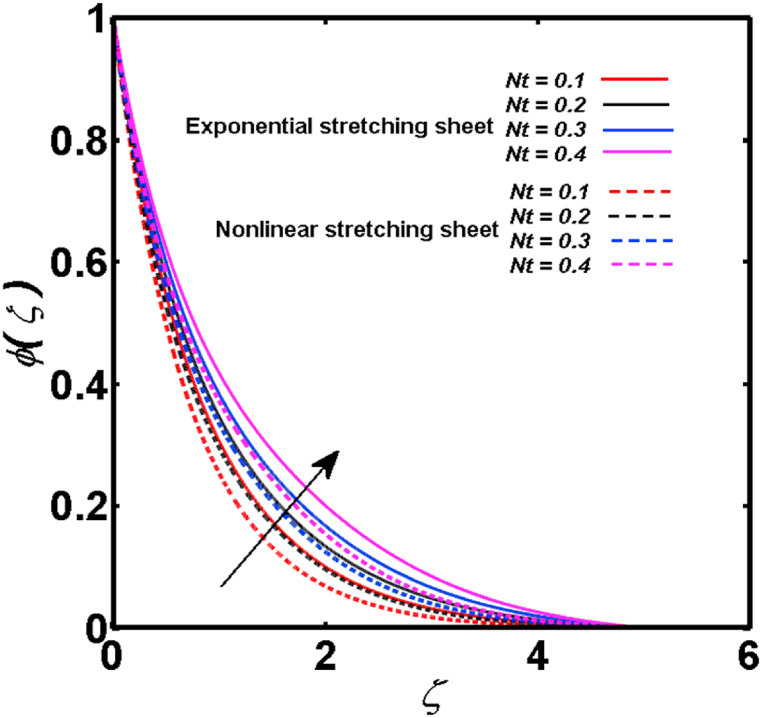
Curve of Φ(ζ) regarding Nt parameter.

**Fig. 20:**
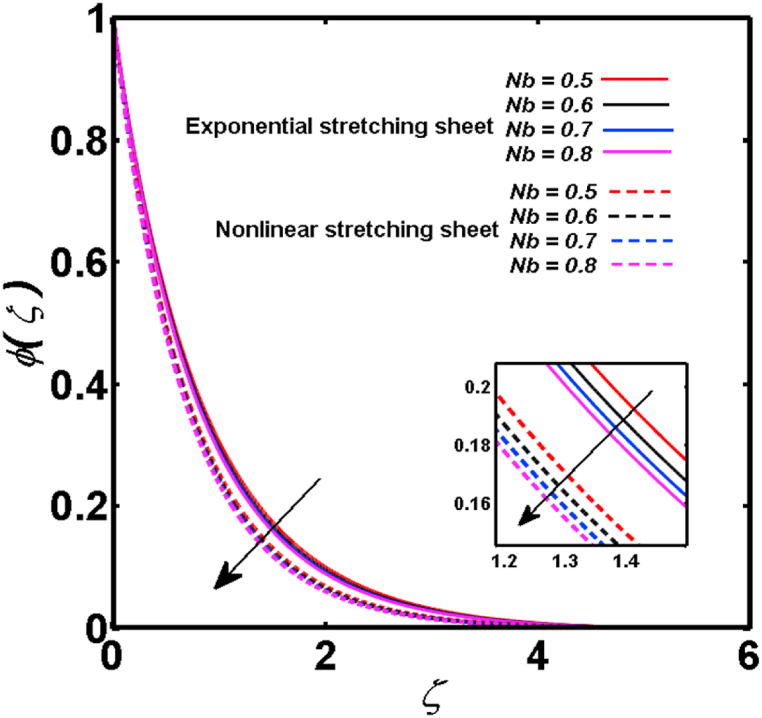
Curve of Φ(ζ) regarding Nb parameter.

An analysis of the 3D steady flow mechanism of Sutterby nanofluid subject to exponential and nonlinear stretchable sheets is carried out in this study. The consequences of the variable magnetic field, partial slips, and variable heat source/sink with the Buongiorno model are included in the heat and mass transfer mechanisms. An effective methodology of bvp4c in MATLAB package is applied to numerically analyze the flow problem. For the accuracy and validation of the ongoing problem, a comparative study has been conducted between the current and previous results in Table 1, Table 2. For both considered sheets, the numerical values in Table 1, Table 2 are in good agreement with the previous study. This comparison exhibits that the current study is accurate and valid for both nonlinear and exponential sheets. Physical visualization of the various features of the flow phenomenon (concentration, velocity, and temperature distributions) relative to the emerging parameters are graphically elucidated. The pertinent parameters are taken in the ranges of $0.5\leq \alpha^{*}\leq 2.0$, 0.1≤Nt≤0.4, $0.5\leq \xi_{2}\leq 2.0$, 3≤Pr≤6, 0.2≤Nb≤0.5, 1≤Re≤4, 2≤De≤8, 0.2≤M≤0.8, $0.5\leq \xi_{1}\leq 2.0$, 0.2≤δ≤0.8, $1.2\leq M^{*}\leq 1.8$. Fig. 3 is prepared to examine the temperature profile for an exponential sheet relative to the greater magnitude of the temperature exponent parameter. Due to the thermal boundary layer's decreasing thickness, the temperature field is reduced by the temperature exponent parameter's escalating values. The pattern of the temperature distribution for both sheets corresponding to the improved thermophoresis parameter is manifested in Fig. 4. The pattern of the temperature field becomes inclining with the higher intensity of the thermophoresis parameter. Physically, thermophoresis is a mechanism in which a small number of nanoparticles move from the hot medium to the cold medium due to the temperature gradient. The improved thermophoresis parameter means the movement of a large number of nanoparticles from the hot area to the cold area with an extremer thermophoretic force. As a result, the temperature profile becomes escalating. For nonlinear and exponential sheets, Fig. 5 portrays the reducing behavior of the temperature curve subject to the improved Prandtl number. The reason is that the fluid thermal diffusivity is inversely related to the Prandtl number. The fluid's thermal diffusivity minimizes due to the augmentation of the Prandtl number. Consequently, the rate of heat distribution within the fluid is lower and the temperature field exhibits diminishing behavior. The consequence of the Brownian motion parameter on the pattern of the temperature distribution is manifested in Fig. 6. The mechanism in which nanoparticles move arbitrarily within the traditional fluid is defined as Brownian motion. With the escalating Brownian motion parameter, the nanoparticle's mobility enhances, and they strongly collide with each other. Accordingly, the kinetic energy of the fluid increased which further augmented the temperature curve for both cases of nonlinear and exponential sheets. In the case of shear thinning behavior of fluid (N<0), the improved Reynolds number diminishes the axial velocity at both nonlinear and exponential sheets as depicted in Fig. 7. On the other hand, for shear thickening fluid (N>0), the axial velocity becomes augmented with the increasing values of Reynolds number in Fig. 8. Physically, the Reynolds number determines the proportion of inertial to viscous forces. The viscous forces diminish with the higher Reynolds number. In the shear-thinning fluid, the viscosity decreases with the higher intensity of the Reynolds number, and accordingly, the flow velocity deteriorates. The reducing viscosity with improved Reynolds number enhances the axial velocity of shear thickening fluid for both nonlinear and exponential sheets. Fig. 9 and Fig. 10 delineate the behavior of axial velocity subject to improved Deborah number for both cases of shear thinning and shear thickening respectively. For shear-thinning fluid, the higher magnitude of the Deborah number decreases the fluid axial velocity. In relation to the Deborah number, the axial velocity is enhanced for shear-thickening fluid. Fig. 11 and Fig. 12 are prepared to scrutinize the accelerating impact of the magnetic field on the distributions of axial and transverse velocities respectively. For both sheets, the stronger magnetic effects decline the curve of the axial velocity as well as transverse velocity. Physically, the enhanced magnetic field develops a resistive Lorentz force which acts in the opposite direction of the fluid flow. As a result, the fluid movement deteriorates, and the thickness of the momentum boundary layer diminishes. Fig. 13 and Fig. 14 are sketched to scrutinize the nature of both velocities (axial and transverse) corresponding to the velocity slip parameters. With the increment in the velocity slip parameters, both the velocity distributions present deteriorating nature. Physically, the velocity slip develops when the fluid velocity and stretching sheet velocity are not equal. Such velocity slip enhances with the improved velocity slip parameter. The momentum boundary layer thickness reduces and consequently, the transverse and axial velocities demonstrate declining behavior. The objective of Fig. 15 and Fig. 16 is to examine the increasing effect of the stretching ratio parameter on the axial and transverse velocities respectively for both exponential and nonlinear sheets. With the higher stretching ratio parameter, there exists an augmentation in the field of transverse velocity. On the other hand, the axial velocity depicts a declining behavior. The physical reason is that the stretching rates along the $\tilde{X}$ and $\tilde{Y}$ directions are related to the stretching ratio parameter. The improved stretching ratio parameter exhibits an inclination in the stretching rate towards the $\tilde{Y}$-direction but lowers the stretching rate towards the $\tilde{X}$ direction. Accordingly, the transverse and axial velocities exhibit increasing and decreasing behavior respectively. The consequence of the power-law index parameter of the nonlinear sheet on the axial and transverse velocities is disclosed in Fig. 17 and Fig. 18 respectively. These graphics demonstrate the declining pattern of the velocities subject to the larger magnitude of the parameter. Fig. 19 discloses the impact of the superior thermophoresis parameter on the concentration distribution. The concentration profile exhibits an inclination with the greater magnitude of the thermophoresis parameter. Fig. 20 portrays the profile of the concentration relying on the accelerating impact of the Brownian motion parameter. With the increased intensity of the Brownian motion parameter, the concentration field develops a diminishing nature. The consequences of the different values of pertinent parameters on local Nusselt number, skin friction coefficients along $\tilde{X}$ and $\tilde{Y}$ directions, and Sherwood number are depicted in Table 3, Table 4. In the case of the nonlinear sheet, the effect of the higher magnitude of the power-law index parameter, Reynolds number, and Deborah number is to develop an enhancement in the Sherwood number and local Nusselt number. The larger amount of these parameters reduces the skin friction coefficients for the nonlinear sheet. For an exponential sheet, the physical quantities exhibit escalating behavior corresponding to the temperature exponent parameter. With the improved Deborah and Reynolds number, the physical quantities show declining behavior for an exponential sheet. The results acquired from this study are useful in applied sciences, electrochemistry, medical sciences, engineering fields, and biological fields.

**Table 1:** For exponential stretching sheet, comparative results of skin friction coefficient towards $\tilde{X}$ and $\tilde{Y}$ directions when $\xi_{1}=\xi_{2}=0$.

	Present	values	Mahanthesh	et al. [33]
δ	$-{\left(\frac{{Re}_{\tilde{X}}}{2}\right)}^{0.5}{Cf}_{\tilde{X}}$	${-\left(\frac{{Re}_{\tilde{Y}}}{2}\right)}^{0.5}{Cf}_{\tilde{Y}}$	$-{\left(\frac{{Re}_{\tilde{X}}}{2}\right)}^{0.5}{Cf}_{\tilde{X}}$	${-\left(\frac{{Re}_{\tilde{Y}}}{2}\right)}^{0.5}{Cf}_{\tilde{Y}}$
0.0	1.28269	0	1.28181	0
0.5	1.57023	0.78511	1.56988	0.78494
1.0	1.81291	1.81291	1.81275	1.81275

**Table 2:** For nonlinear stretching sheet, comparative results of skin friction coefficient towards $\tilde{X}$ and $\tilde{Y}$ directions when $M^{*}=1$.

	Present	values	Mahanthesh	et al. [34]
δ	$-{\left({Re}_{\tilde{X}}\right)}^{0.5}{Cf}_{\tilde{X}}$	${-\left({Re}_{\tilde{Y}}\right)}^{0.5}{Cf}_{\tilde{Y}}$	$-{\left({Re}_{\tilde{X}}\right)}^{0.5}{Cf}_{\tilde{X}}$	${-\left({Re}_{\tilde{Y}}\right)}^{0.5}{Cf}_{\tilde{Y}}$
0.0	1	0	1	0
0.5	1.22526	0.61263	1.22474	0.61237
1.0	1.41444	1.41444	1.41421	1.41421

**Table 3:** For nonlinear sheet, numerical values of physical quantities relative to different parameters.

Re	De	$M^{*}$	${\left({Re}_{\tilde{X}}\right)}^{0.5}{Cf}_{\tilde{X}}$	${{Re}_{\tilde{Y}}}^{0.5}{Cf}_{\tilde{Y}}$	${\left({Re}_{\tilde{X}}\right)}^{-0.5}{Nu}_{\tilde{X}}$	${\left({Re}_{\tilde{X}}\right)}^{-0.5}{Sh}_{\tilde{X}}$
1.0 2.0 3.0	2.0	1.2	−0.77972887	−0.38694784	0.94103639	1.4295555
			−0.78820496	−0.38839754	0.94414565	1.4339037
			−0.79640644	−0.38982574	0.94711871	1.4380765
	1.5		−0.77756374	−0.38658187	0.94023612	1.4284389
	2.5		−0.78187510	−0.38731237	0.94182724	1.4306599
	3.5		−0.7861127	−0.38803719	0.94338164	1.4328338
		1.2	−0.77972887	−0.38694784	0.94103639	1.4295555
		1.4	−0.79975968	−0.39670478	1.0137420	1.5182600
		1.6	−0.81809517	−0.40562133	1.0803576	1.6006687

**Table 4:** For exponential sheet, numerical values of physical quantities relative to different parameters.

Re	De	$\alpha^{*}$	${\left(\frac{{Re}_{\tilde{X}}}{2}\right)}^{0.5}{Cf}_{\tilde{X}}$	${\left(\frac{{Re}_{\tilde{Y}}}{2}\right)}^{0.5}{Cf}_{\tilde{Y}}$	${\left(\frac{2}{{Re}_{\tilde{X}}}\right)}^{0.5}{Nu}_{\tilde{X}}$	${\left(\frac{2}{{Re}_{\tilde{X}}}\right)}^{0.5}{Sh}_{\tilde{X}}$
1.0 2.0 3.0	2.0	0.5	−0.83641581	−0.41300063	0.56577648	1.0128239
			−0.85001429	−0.41438436	0.56409311	1.0107741
			−0.86405750	−0.41576537	0.56236271	1.0086710
	1.5		−0.83308183	−0.41265437	0.56619033	1.0133284
	2.5		−0.83977555	−0.41334677	0.56535988	1.0123163
	3.5		−0.84657430	−0.41403865	0.56451824	1.0112914
		0.5	−0.83641581	−0.41300063	0.56577648	1.0128239
		1.0	−0.83641564	−0.41300055	0.79610973	1.2832948
		1.5	−0.83641544	−0.41300034	0.98870948	1.5238007

## 6 Conclusion

An exploration of the boundary layer time-independent flow phenomenon of a Sutterby nanofluid subject to shear thinning and shear thickening behavior is carried out in this study. The main findings of this study are illustrated as follows.

• The accelerating values of Reynolds and Deborah numbers decline the pattern of the axial velocity in the case of the shear thinning fluid.
• Both the transverse and axial velocities present a declining nature with the augmented magnetic field and velocity slip parameters.
• The power-law index parameter of the nonlinear sheet with escalating magnitude lowers the velocities (transverse and axial).
• The objective of the improved temperature exponent parameter is to develop a decrement in the temperature field.
• The concentration field exhibits a declining pattern relative to the Brownian motion but presents an increment with the higher values of the thermophoresis parameter.
• The current flow phenomenon can be analyzed for various non-Newtonian fluid models with different physical effects involving inclined magnetic field, thermal radiation, viscous dissipation, chemical reactions, and Cattaneo-Christov heat and mass flux theories.

## Author contribution statement

Bushra Ishtiaq: Conceived and designed the analysis; Wrote the paper.

Sohail Nadeem: Conceived and designed the analysis; Analyzed and interpreted the data.

Jehad Alzabut: Contributed analysis tools or data.

## Data availability statement

Data will be made available on request.

## Declaration of competing interest

The corresponding author on behalf of all authors declares “no conflict of interest” with anyone exists.

## References

[1] Sutterby J.L. (1996). Laminar converging flow of dilute polymer solutions in conical sections: Part I. Viscosity data, new viscosity model, tube flow solution. AIChE J..

[2] Ahmad S., Farooq M., Javed M., Anjum A. (2018). Double stratification effects in chemically reactive squeezed Sutterby fluid flow with thermal radiation and mixed convection. Results Phys..

[3] Bouslimi J., Alkathiri A.A., Alharbi A.N., Jamshed W., Eid M.R., Bouazizi M.L. (2022). Dynamics of convective slippery constraints on hybrid radiative Sutterby nanofluid flow by Galerkin finite element simulation. Nanotechnol. Rev..

[4] Shahzad F., Bouslimi J., Gouadria S., Jamshed W., Eid M.R., Safdar R., Shamshuddin M.D., Nisar K.S. (2022). Hydrogen energy storage optimization in solar-HVAC using Sutterby nanofluid via Koo-Kleinstreuer and Li (KKL) correlations model: a solar thermal application. Int. J. Hydrog..

[5] Jamshed W., Eid M.R., Safdar R., Pasha A.A., Mohamed Isa S.S., Adil M., Rehman Z., Weera W. (2022). Solar energy optimization in solar-HVAC using Sutterby hybrid nanofluid with Smoluchowski temperature conditions: a solar thermal application. Sci. Rep..

[6] Bouslimi J., Alkathiri A.A., Althagafi T.M., Jamshed W., Eid M.R. (2022). Thermal properties, flow and comparison between Cu and Ag nanoparticles suspended in sodium alginate as Sutterby nanofluids in solar collector. Case Stud. Therm. Eng..

[7] Abdal S., Siddique I., Afzal S., Sharifi S., Salimi M., Ahmadian A. (2022). An analysis for variable physical properties involved in the nano-biofilm transportation of Sutterby fluid across shrinking/stretching surface. Nanomaterials.

[8] Rehman M.I., Chen H., Hamid A., Jamshed W., Eid M.R., El Din S.M., Khalifa H.A., Abd-Elmonem A. (2023). Effect of Cattaneo-Christov heat flux case on Darcy-Forchheimer flowing of Sutterby nanofluid with chemical reactive and thermal radiative impacts. Case Stud. Therm. Eng..

[9] Buongiorno J. (2006). Convective transport in nanofluids.

[10] Khan U., Zaib A., Shah Z., Baleanu D., Sherif E.S. (2020). Impact of magnetic field on boundary-layer flow of Sisko liquid comprising nanomaterials migration through radially shrinking/stretching surface with zero mass flux. J. Mater. Res. Technol..

[11] Gangadhar K., Kumari M.A., Chamkha A.J. (2022). EMHD flow of radiative second-grade nanofluid over a Riga Plate due to convective heating: revised Buongiorno's nanofluid model. Arabian J. Sci. Eng..

[12] Gangadhar K., Kumari M.A., Venkata Subba Rao M., Chamkha A.J. (2022). Oldroyd-B nanoliquid flow through a triple stratified medium submerged with gyrotactic bioconvection and nonlinear radiations. Arabian J. Sci. Eng..

[13] Ishtiaq B., Nadeem S. (2022).

[14] Gangadhar K., Edukondala Nayak R., Venkata Subba Rao M., Chamkha A.J. (2022). Nonlinear radiations in chemically reactive Walter's B nanoliquid flow through a rotating cone. Proc. Inst. Mech. Eng.: J. Process Mech. Eng..

[15] Yasir M., Hafeez A., Khan M. (2022). Thermal conductivity performance in hybrid (SWCNTs-CuO/Ehylene glycol) nanofluid flow: dual solutions. Ain Shams Eng. J..

[16] Ishtiaq B., Zidan A.M., Nadeem S., Alaoui M.K. (2022). Analysis of entropy generation in the nonlinear thermal radiative micropolar nanofluid flow towards a stagnation point with catalytic effects. Phys. Scripta.

[17] Yasir M., Malik Z.U., Alzahrani A.K., Khan M. (2022).

[18] Nadeem S., Tumreen M., Ishtiaq B., Abbas N. (2022).

[19] Gangadhar K., Mary Victoria E., Chamkha A.J. (2022).

[20] Nadeem S., Ishtiaq B., Abbas N. (2023). Impact of thermal radiation on two-dimensional unsteady third-grade fluid flow over a permeable stretching Riga plate. Int. J. Mod. Phys. B.

[21] Khan U., Zaib A., Ishak A., Waini I., Pop I., Elattar S., Abed A.M. (2023). Stagnation point flow of a water-based graphene-oxide over a stretching/shrinking sheet under an induced magnetic field with homogeneous-heterogeneous chemical reaction. J. Magn. Magn Mater..

[22] Gangadhar K., Edukondala Nayak R., Venkata Subba Rao M., Kannan T. (2021). Nodal/Saddle stagnation point slip flow of an aqueous convectional magnesium oxide–gold hybrid nanofluid with viscous dissipation. Arabian J. Sci. Eng..

[23] Wang F., Kumar R.N., Prasannakumara B.C., Khan U., Zaib A., Abdel-Aty A.H., Yahia I.S., Alqahtani M.S., Galal A.M. (2022). Aspects of uniform horizontal magnetic field and nanoparticle aggregation in the flow of nanofluid with melting heat transfer. Nanomaterials.

[24] Sajid T., Jamshed W., Shahzad F., Akgül E.K., Nisar K.S., Eid M.R. (2022). Impact of gold nanoparticles along with Maxwell velocity and Smoluchowski temperature slip boundary conditions on fluid flow: Sutterby model. Chin. J. Phys..

[25] Khan U., Zaib A., Ishak A., Eldin S.M., Alotaibi A.M., Raizah Z., Waini I., Abed S.A.M. (2023). Features of hybridized AA7072 and AA7075 alloys nanomaterials with melting heat transfer past a movable cylinder with Thompson and Troian slip effect. Arab. J. Chem..

[26] Bhargavi D.N., Gangadhar K., Chamkha A.J. (2022). Graphene-gold/PDMS Maxwell hybrid nanofluidic flow in a squeezed channel with linear and irregular radiations. Proc. Inst. Mech. Eng.: J. Process Mech. Eng..

[27] Gangadhar K., Manasa Seshakumari P., Venkata Subba Rao M., Chamkha A.J. (2022). Biconvective transport of magnetized couple stress fluid over a radiative paraboloid of revolution. Proc. Inst. Mech. Eng.: J. Process Mech. Eng..

[28] Gangadhar K., Bhanu Lakshmi K., El-Sapa S., Venkata Subba Rao M., Chamkha A.J. (2022).

[29] Gangadhar K., Chamkha A.J. (2021). Entropy minimization on magnetized Boussinesq couple stress fluid with non-uniform heat generation. Phys. Scripta.

[30] Gangadhar K., Bhanu Lakshmi K., Kannan T., Chamkha A.J. (2022).

[31] Kotha G., Kolipaula V.R., Venkata Subba Rao M., Penki S., Chamkha A.J. (2020). Internal heat generation on bioconvection of an MHD nanofluid flow due to gyrotactic microorganisms. Eur. Phys. J. Plus..

[32] Zainal N.A., Nazar R., Naganthran K., Pop I. (2021). Heat generation/absorption effect on MHD flow of hybrid nanofluid over bidirectional exponential stretching/shrinking sheet. Chin. J. Phys..

[33] Mahanthesh B., Mabood F., Gireesha B.J., Gorla R.S. (2017). Effects of chemical reaction and partial slip on the three-dimensional flow of a nanofluid impinging on an exponentially stretching surface. Eur. Phys. J. Plus..

[34] Mahanthesh B., Gireesha B.J., Gorla R.S., Makinde O.D. (2018). Magnetohydrodynamic three-dimensional flow of nanofluids with slip and thermal radiation over a nonlinear stretching sheet: a numerical study. Neural Comput. Appl..

[35] Amirsom N.A., Uddin M.J., Basir M.F.M., Ismail A.I.M., Beg O.A., Kadir A. (2019). Three-dimensional bioconvection nanofluid flow from a bi-axial stretching sheet with anisotropic slip. Sains Malays..

